# Supplementary material for: High-Fat Diet Induces Pre-Diabetes and Distinct Sex-Specific Metabolic Alterations in Negr1-Deficient Mice
Source: Biomedicines. 2021 Sep 3;9(9):1148. doi: 10.3390/biomedicines9091148 (PMC8466019; doi:10.3390/biomedicines9091148)

Supplementary figures:

Maria Kaare, Kaie Mikheim, Kersti Lilleväli, Kalle Kilk, Toomas Jagomäe, Este Leidmaa, Maria Piirsalu, Rando Porosk, Katyayani Singh, Riin Reimets, Egon Taalberg, Michael K. E. Schäfer, Mario Plaas, Eero Vasar and Mari-Anne Philips

High fat diet induces prediabetes and distinct metabolic alterations sex-specifically in Negr1 deficient mice

Supplementary figure S1: The time graphs of food preference test. **p≥0.01, ***p≥0.001 (Mann-Withney U test)

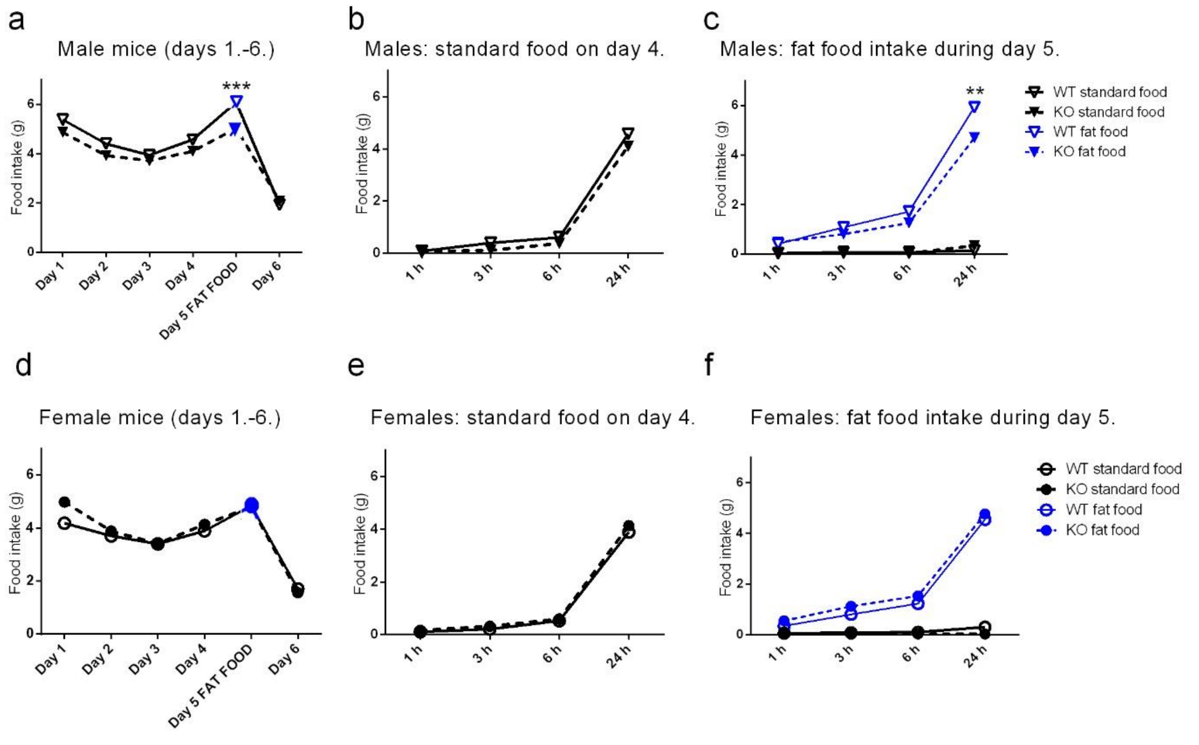

Supplementary figure S2: Organic acids. The levels of selected organic acids in males, females and in both sexes combined. Data represent mean±SEM, *p≥0.05, two-way ANOVA (Bonferroni *post hoc* test).

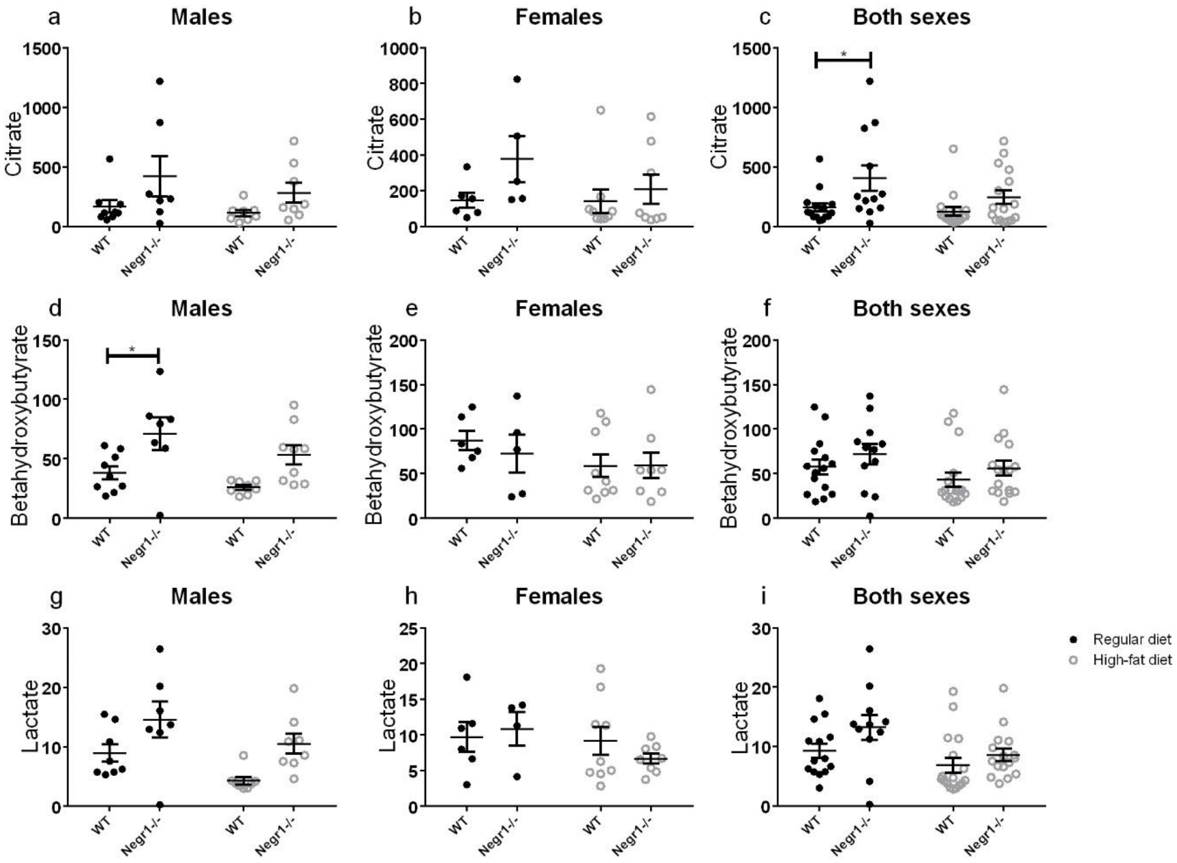

Supplementary figure S3: BODIPY stainings of male livers. BODIPY staining (green) of livers from individual mice in (a-j) standard and (k-s) high-fat diet treatment group. Columns (a-e, k-o) present analyzed WT and (f-j, p-s) Negr1-deficient mice, respectively. Neutral lipid droplets of (a-e) WT mice liver undergoing a standard diet. (f-j) Negr1-deficient mice in a standard diet display increased neutral lipid content in the hepatocytes. High-fat diet leads to an increase in hepatocytic lipid droplets in (k-o) WT and seems to have a minuscule effect on (p-s) Negr1-/- mice. The male mice used for liver stainings were 5 months old. Nuclei (blue) were stained using H33258 stain. Scale bar: 100 µm.

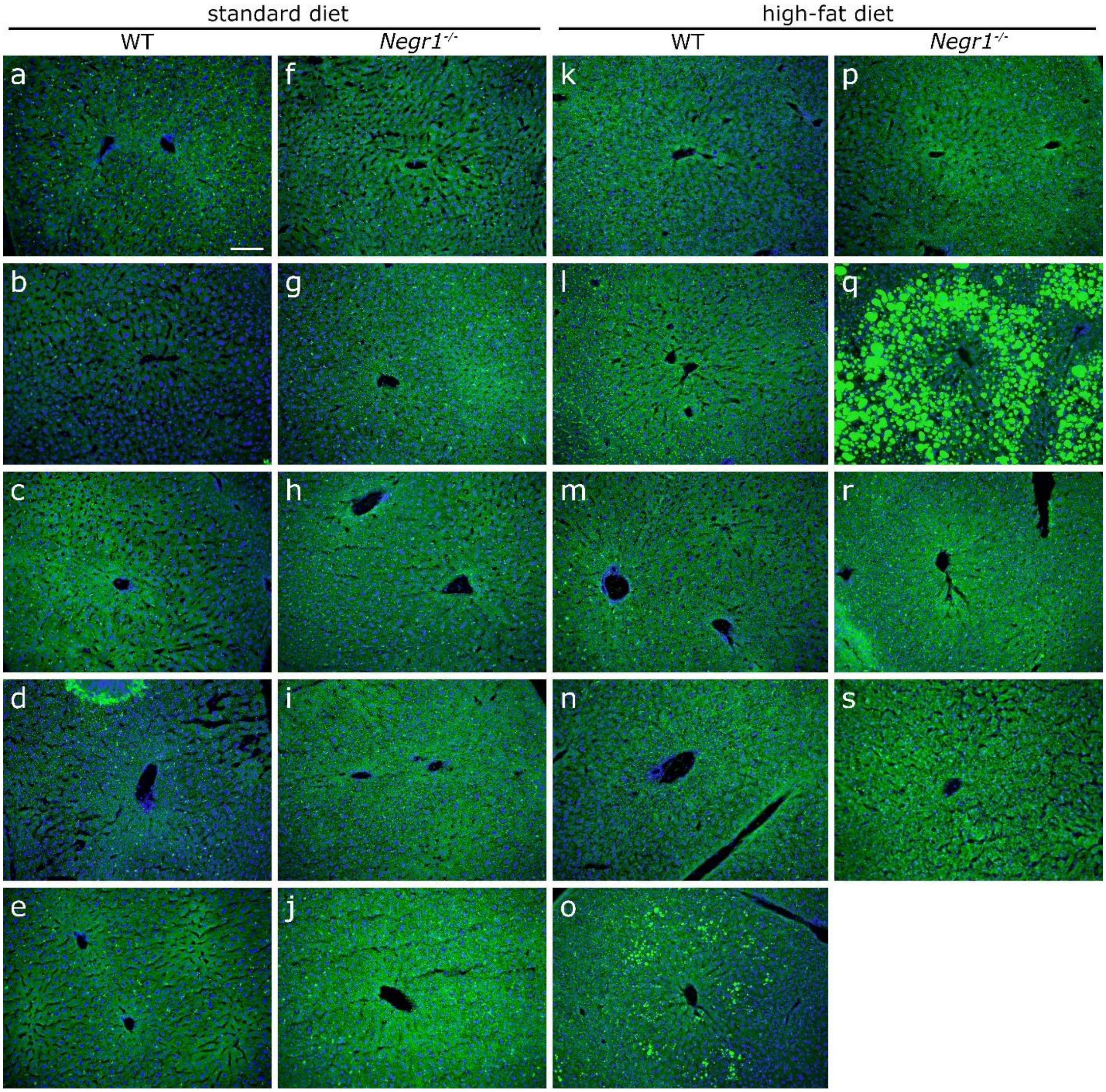

Supplementary figure S4: BODIPY staining of female livers. BODIPY staining (green) of individual mice in (a-h) standard and (i-q) high-fat treatment groups. Columns (a-c, i-l) present analyzed WT and (d-h, m-q) *Negr1*$^{-/-}$ mice, respectively. High-fat diet group received high-fat food for 9 weeks. The age of the female mice used for liver stainings were 8-9 months. Nuclei (blue) were stained using H33258 stain. Scale bar: 100 µm.

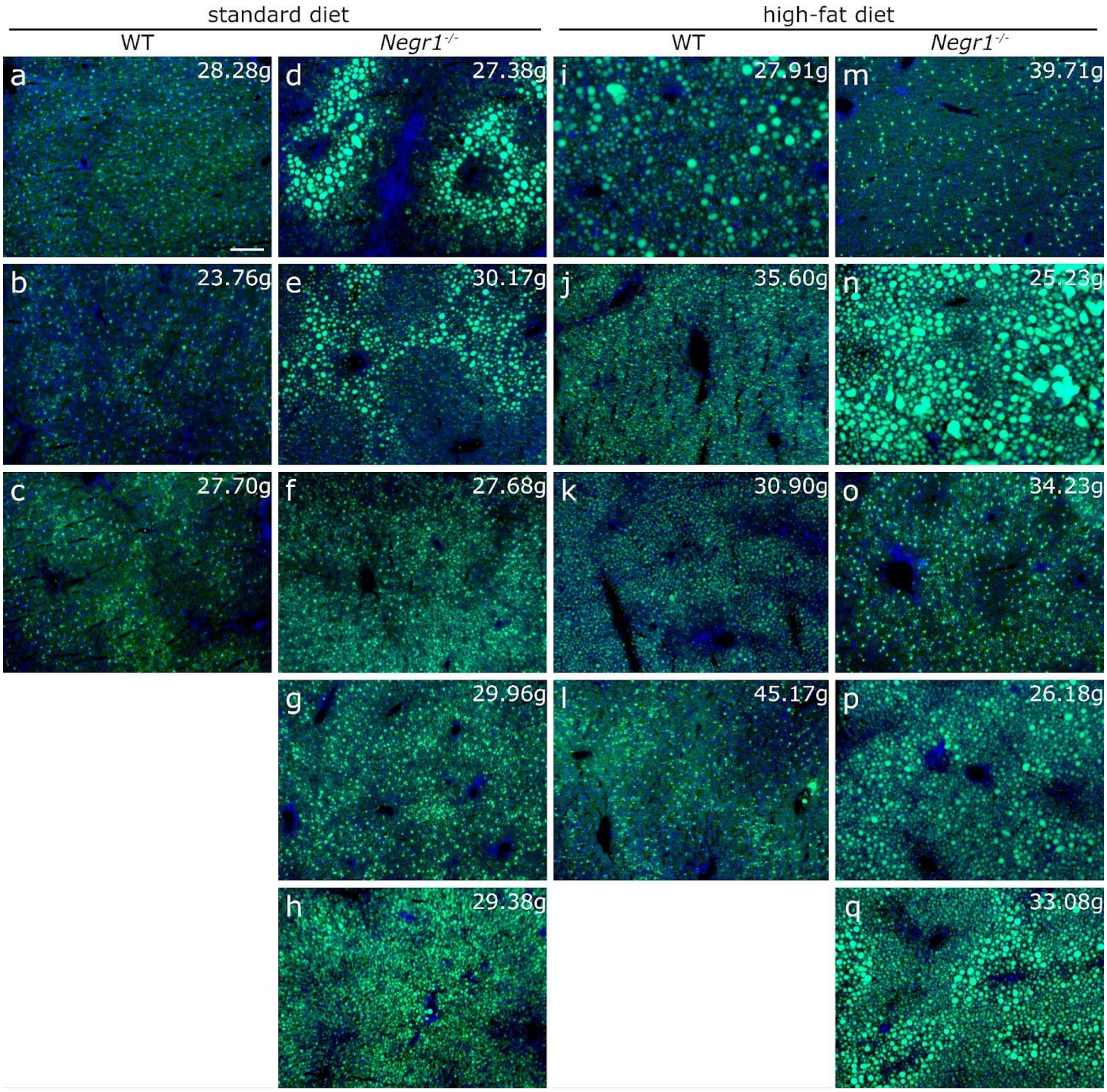

Supplementary figure S5: **BODIPY stainings of female livers**. Representative images of BODIPY neutral lipid staining (green) from (a, c) WT and (b, d) *Negr1*$^{-/-}$ liver sections that underwent (a-b) standard and (c-d) high-fat diet. Compared to (a) WT mice, the hepatocytes from (b) *Negr1*$^{-/-}$ mice on the standard diet display increased lipid droplet accumulation in the liver. This genotype dependent difference is diminished with (c, d) high-fat diet treatment. The age of the mice used for liver stainings were around 8-9 months. Nuclei (blue) were stained using H33258 stain. Scale bars: 100 µm, 25 µm (inserts).

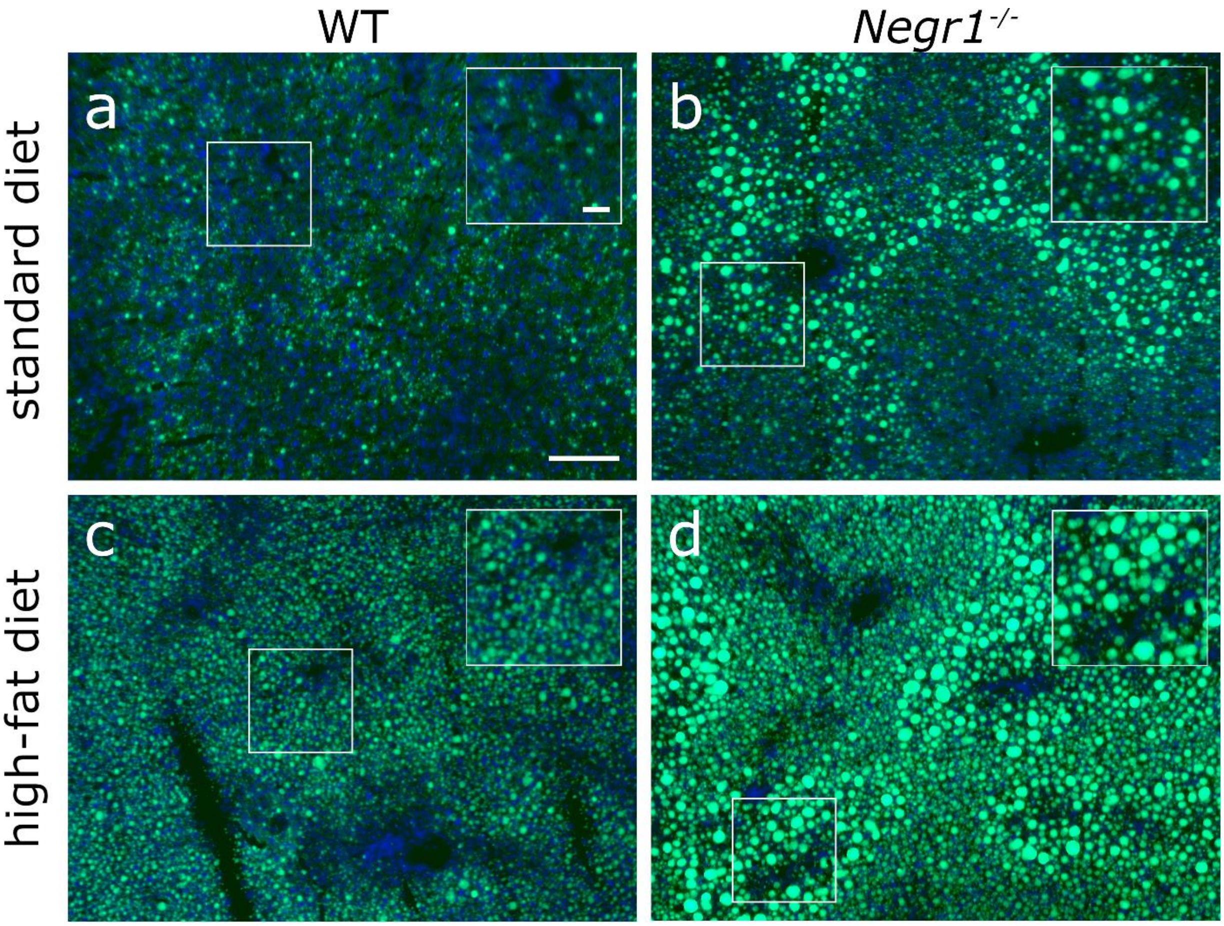

Supplementary figure S6: Cross-sectional area of muscle fibers from female mice. Phalloidin (red) stained cryosections from the quadriceps femoris muscle (a, d) WT and (b, e) Negr1-/- mice sections underwent (a-c) standard and (d-f) high-fat diets. Of Negr1−/− (b and e) and wild type (a and d) mice receiving standard (a and b) or high fat (d and e) diets. (c, f) morphometric measurements of muscle fiber cross-sectional area revealed no statistically significant changes between groups. The age of female mice used for muscle stainings were around 8-9 months. Nuclei (blue) were stained using H33258 stain. Scale bar: 100 µm.

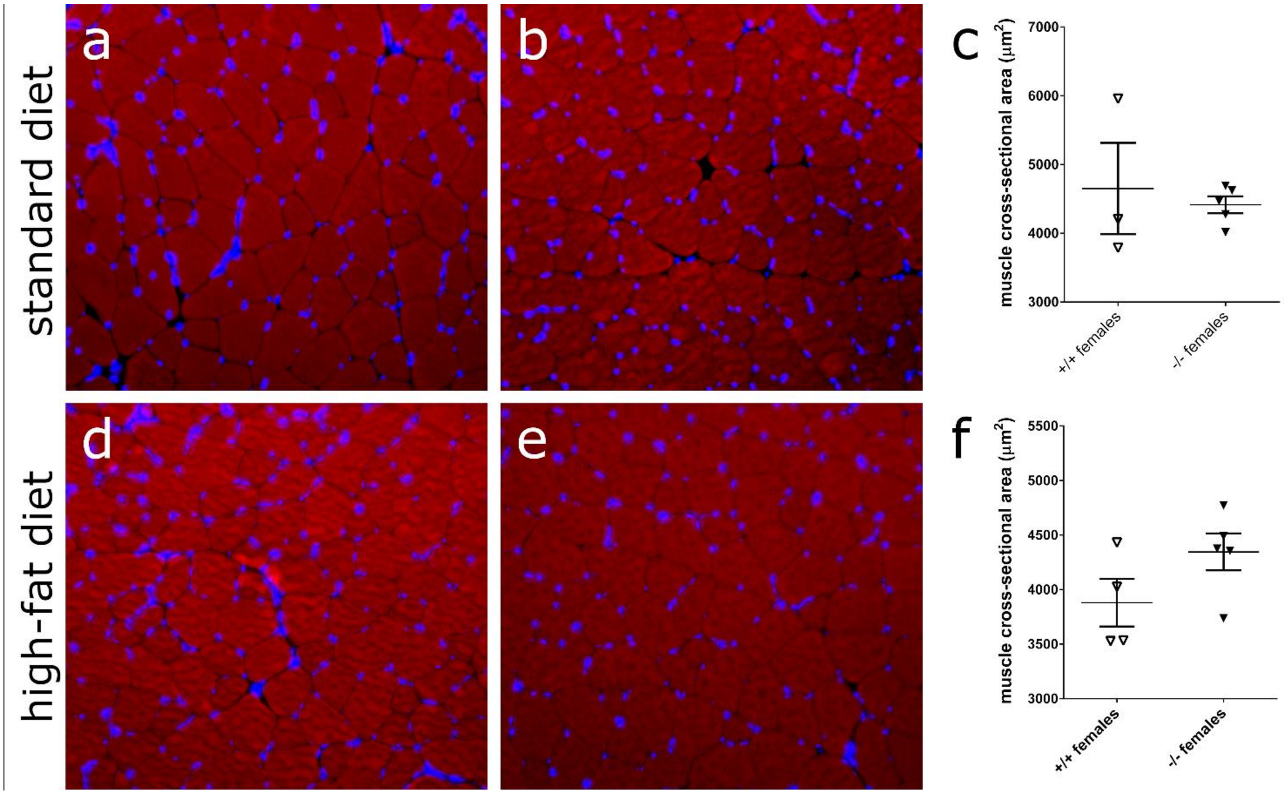

Supplement: Supplementary file 1 [file biomedicines-09-01148-s001.zip › Supplementary figures.pdf]
