# Supplementary material for: High-Fat Diet Induces Pre-Diabetes and Distinct Sex-Specific Metabolic Alterations in Negr1-Deficient Mice
Source: Biomedicines. 2021 Sep 3;9(9):1148. doi: 10.3390/biomedicines9091148 (PMC8466019; doi:10.3390/biomedicines9091148)
Supplement: Supplementary file 1 [file biomedicines-09-01148-s001.zip › Supplementary table S1.pdf]

Metabolites	Sex	Normal diet		HFD		Two-way ANOVA		
		**Wild type** **Mean** ± SEM	**Negr1-/-** **Mean** ± SEM	**Wild type** **Mean** ± SEM	**Negr1-/-** **Mean** ± SEM		***F***	**p**
**Acylcarnitines**								
Carnitine (C0)	Male	21.4 ± 1.1	20.7 ± 1.3	14.1 ± 0.7	14.0 ± 1.1	**Diet**	43.95	**<0.0001**
						**Genotype**	0.125	0.7258
						**Diet X genotype**	0.078	0.7826
	Female	17.7 ± 1.3	17.1 ± 1.0	11.5 ± 0.8	9.3 ± 0.5	**Diet**	53.14	**<0.0001**
						**Genotype**	1.986	0.1676
						**Diet X genotype**	0.636	0.4305
Acetylcarnitine (C2)	Male	11.3 ± 0.9	12.4 ± 0.9	6.1 ± 0.3	6.6 ± 0.5	**Diet**	59.11	**<0.0001**
						**Genotype**	1.055	0.3112
						**Diet X genotype**	0.163	0.6887
	Female	15.2 ± 1.3	10.7 ± 0.8	4.9 ± 0.2	4.2 ± 0.3	**Diet**	118.7	**<0.0001**
						**Genotype**	11.73	**0.0016**
						**Diet X genotype**	6.421	**0.0159**
Proprionyl-carnitine (C3)	Male	0.4 ± 0.02	0.5 ± 0.04	0.3 ± 0.03	0.3 ± 0.02	**Diet**	30.95	**<0.0001**
						**Genotype**	8.642	**0.0057**
						**Diet X genotype**	3.020	0.0908
	Female	0.5 ± 0.04	0.5 ± 0.04	0.2 ± 0.01	0.2 ± 0.02	**Diet**	78.78	**<0.0001**
						**Genotype**	0.823	0.3705
						**Diet X genotype**	0.042	0.8390
Hydroxybuturyl-carnitine (C3-DC)	Male	0.08 ± 0.006	0.09 ± 0.008	0.07 ± 0.009	0.08 ± 0.007	**Diet**	3.115	0.0860
						**Genotype**	1.709	0.1993
						**Diet X genotype**	0.017	0.8967
	Female	0.09 ± 0.01	0.07 ± 0.005	0.06 ± 0.005	0.06 ± 0.006	**Diet**	8.097	**0.0074**
						**Genotype**	0.247	0.6226
						**Diet X genotype**	0.565	0.4573
Hydroxyproprionyl-carnitine (C3-OH)	Male	0.06 ± 0.03	0.05 ± 0.006	0.05 ± 0.006	0.06 ± 0.005	**Diet**	0.313	0.5793
						**Genotype**	0.040	0.8433
						**Diet X genotype**	2.508	0.1220
	Female	0.04 ± 0.004	0.04 ± 0.005	0.06 ± 0.005	0.05 ± 0.005	**Diet**	8.410	**0.0064**
						**Genotype**	0.698	0.4092

						**Diet X genotype**	0.660	0.4222
Butyryl- and isobutyrylcarnitine (C4-)	Male	0.6 ± 0.09	0.6 ± 0.04	0.4 ± 0.04	0.4 ± 0.03	**Diet**	16.09	**0.0003**
						**Genotype**	0.002	0.9641
						**Diet X genotype**	0.2796	0.6002
	Female	0.6 ± 0.05	0.5 ± 0.03	0.3 ± 0.02	0.3 ± 0.01	**Diet**	58.70	**<0.0001**
						**Genotype**	2.943	0.0951
						**Diet X genotype**	0.0007	0.9323
Propenoylcarnitine (C3:1)	Male	0.04 ± 0.003	0.05 ± 0.002	0.04 ± 0.005	0.05 ± 0.005	**Diet**	0.169	0.6831
						**Genotype**	3.923	0.0553
						**Diet X genotype**	1.167	0.2872
	Female	0.04 ± 0.004	0.04 ± 0.004	0.05 ± 0.006	0.05 ± 0.005	**Diet**	1.136	0.2939
						**Genotype**	0.095	0.7595
						**Diet X genotype**	0.154	0.6970
Butenoylcarnitine (C4:1)	Male	0.06 ± 0.003	0.07 ± 0.009	0.06 ± 0.004	0.07 ± 0.006	**Diet**	0.492	0.4875
						**Genotype**	1.461	0.2346
						**Diet X genotype**	0.032	0.8601
	Female	0.06 ± 0.005	0.05 ± 0.003	0.06 ± 0.004	0.07 ± 0.006	**Diet**	9.094	**0.0048**
						**Genotype**	0.184	0.6709
						**Diet X genotype**	4.187	**0.0483**
Isovalerylcarnitine and 2-methybutyrylcarnitine (C5-)	Male	0.2 ± 0.01	0.2 ± 0.01	0.2 ± 0.02	0.2 ± 0.02	**Diet**	0.596	0.4452
						**Genotype**	0.146	0.7042
						**Diet X genotype**	0.606	0.4415
	Female	0.2 ± 0.03	0.2 ± 0.01	0.2 ± 0.01	0.2 ± 0.02	**Diet**	5.922	**0.0202**
						**Genotype**	1.089	0.3038
						**Diet X genotype**	0.224	0.6390
Glutarylcarnitine (C5-DC)	Male	0.04 ± 0.004	0.03 ± 0.004	0.03 ± 0.003	0.04 ± 0.004	**Diet**	0.024	0.8789
						**Genotype**	0.0006	0.9797
						**Diet X genotype**	5.777	**0.0215**
	Female	0.04 ± 0.006	0.03 ± 0.005	0.03 ± 0.003	0.03 ± 0.004	**Diet**	0.599	0.4442
						**Genotype**	0.409	0.5266
						**Diet X genotype**	0.002	0.9629
Hydroxyvaleryl-carnitine (C5:1)	Male	0.1 ± 0.01	0.1 ± 0.006	0.1 ± 0.008	0.1 ± 0.008	**Diet**	1.190	0.2826
						**Genotype**	0.088	0.7686
						**Diet X genotype**	3.699	0.0624

	Female	0.1 ± 0.01	0.1 ± 0.007	0.1 ± 0.008	0.1 ± 0.005	**Diet**	4.018	0.0528
						**Genotype**	6.759	**0.0136**
						**Diet X genotype**	0.059	0.8101
Tiglylcarnitine (C5:1)	Male	0.05 ± 0.003	0.06 ± 0.003	0.05 ± 0.005	0.06 ± 0.005	**Diet**	0.139	0.7117
						**Genotype**	2.851	0.0999
						**Diet X genotype**	0.002	0.9607
	Female	0.05 ± 0.005	0.06 ± 0.005	0.06 ± 0.003	0.06 ± 0.006	**Diet**	0.714	0.4037
						**Genotype**	1.238	0.2732
						**Diet X genotype**	0.061	0.8061
Glutaconylcarnitine (C5:1-DC)	Male	0.04 ± 0.003	0.04 ± 0.004	0.04 ± 0.004	0.05 ± 0.004	**Diet**	2.943	0.0948
						**Genotype**	1.884	0.1784
						**Diet X genotype**	3.221	0.0811
	Female	0.05 ± 0.005	0.04 ± 0.004	0.05 ± 0.004	0.05 ± 0.003	**Diet**	0.024	0.8790
						**Genotype**	1.689	0.2022
						**Diet X genotype**	1.883	0.1787
Hexanoylcarnitine (C6)	Male	0.02 ± 0.002	0.07 ± 0.02	0.02 ± 0.002	0.02 ± 0.002	**Diet**	3.985	0.0535
						**Genotype**	4.204	0.0477
						**Diet X genotype**	3.669	0.0634
	Female	0.04 ± 0.02	0.02 ± 0.002	0.02 ± 0.001	0.02 ± 0.001	**Diet**	2.242	0.1433
						**Genotype**	1.879	0.1792
						**Diet X genotype**	1.146	0.2917
Hexenoylcarnitine (C6:1)	Male	0.01 ± 0.0005	0.01 ± 0.001	0.01 ± 0.0007	0.01 ± 0.001	**Diet**	2.528	0.1206
						**Genotype**	1.784	0.1901
						**Diet X genotype**	2.949	0.0945
	Female	0.01 ± 0.0008	0.01 ± 0.001	0.01 ± 0.001	0.01 ± 0.001	**Diet**	2.147	0.2807
						**Genotype**	0.068	0.7962
						**Diet X genotype**	1.200	0.2807
Pimelylcarnitine (C7-DC)	Male	0.03 ± 0.003	0.03 ± 0.002	0.02 ± 0.002	0.02 ± 0.002	**Diet**	6.114	**0.0183**
						**Genotype**	0.078	0.7815
						**Diet X genotype**	3.033	0.0901
	Female	0.02 ± 0.003	0.02 ± 0.002	0.02 ± 0.004	0.02 ± 0.003	**Diet**	0.462	0.5014
						**Genotype**	0.368	0.5483
						**Diet X genotype**	0.753	0.3916

Octanoylcarnitine (C8)	Male	0.2 ± 0.008	0.2 ± 0.01	0.2 ± 0.008	0.2 ± 0.006	**Diet**	2.489	0.1234
						**Genotype**	0.726	0.3997
						**Diet X genotype**	0.070	0.7926
	Female	0.2 ± 0.007	0.2 ± 0.009	0.2 ± 0.008	0.1 ± 0.006	**Diet**	0.005	0.9459
						**Genotype**	0.054	0.8173
						**Diet X genotype**	3.324	0.0768
Nonanoylcarnitine (C9)	Male	0.06 ± 0.003	0.05 ± 0.004	0.06 ± 0.006	0.05 ± 0.005	**Diet**	0.610	0.4399
						**Genotype**	1.421	0.2411
						**Diet X genotype**	0.015	0.9025
	Female	0.06 ± 0.007	0.05 ± 0.005	0.05 ± 0.005	0.06 ± 0.006	**Diet**	0.134	0.7167
						**Genotype**	0.293	0.5920
						**Diet X genotype**	0.646	0.4271
Decanoylcarnitine (C10)	Male	0.1 ± 0.006	0.2 ± 0.01	0.16 ± 0.015	0.18 ± 0.01	**Diet**	0.605	0.4418
						**Genotype**	7.033	**0.0118**
						**Diet X genotype**	0.064	0.8017
	Female	0.2 ± 0.005	0.2 ± 0.008	0.16 ± 0.007	0.17 ± 0.01	**Diet**	0.631	0.4324
						**Genotype**	0.906	0.3476
						**Diet X genotype**	0.105	0.7483
Decenoylcarnitine (C10:1)	Male	0.07 ± 0.006	0.07 ± 0.005	0.06 ± 0.006	0.08 ± 0.004	**Diet**	0.190	0.6659
						**Genotype**	2.336	0.1351
						**Diet X genotype**	1.020	0.3194
	Female	0.08 ± 0.005	0.07 ± 0.005	0.07 ± 0.007	0.06 ± 0.005	**Diet**	2.160	0.1505
						**Genotype**	5.226	**0.0284**
						**Diet X genotype**	0.249	0.6211
Decadienyl-carnitine (C10:2)	Male	0.03 ± 0.002	0.03 ± 0.002	0.03 ± 0.003	0.03 ± 0.004	**Diet**	0.782	0.3823
						**Genotype**	2.108	0.1552
						**Diet X genotype**	1.250	0.2709
	Female	0.04 ± 0.004	0.03 ± 0.003	0.04 ± 0.006	0.03 ± 0.004	**Diet**	0.112	0.7394
						**Genotype**	4.488	**0.0413**
						**Diet X genotype**	0.046	0.8323
Dodecanoyl-carnitine (C12)	Male	0.07 ± 0.005	0.08 ± 0.004	0.07 ± 0.005	0.07 ± 0.007	**Diet**	1.522	0.2253
						**Genotype**	0.843	0.3647
						**Diet X genotype**	0.772	0.3855
	Female	0.07 ± 0.003	0.06 ± 0.007	0.06 ± 0.006	0.05 ± 0.004	**Diet**	2.779	0.1044

						**Genotype**	2.959	0.0942
						**Diet X genotype**	0.007	0.9353
Dodecanedioyl-carnitine (C12-DC)	Male	0.09 ± 0.008	0.09 ± 0.006	0.10 ± 0.004	0.09 ± 0.008	**Diet**	0.876	0.3555
						**Genotype**	0.530	0.4713
						**Diet X genotype**	0.530	0.4713
	Female	0.08 ± 0.006	0.09 ± 0.005	0.10 ± 0.008	0.08 ± 0.007	**Diet**	0.590	**0.0367**
						**Genotype**	0.009	0.9263
						**Diet X genotype**	4.717	0.4477
Dodecenoyl-Carnitine (C12:1)	Male	0.6 ± 0.005	0.05 ± 0.005	0.05 ± 0.004	0.06 ± 0.004	**Diet**	0.018	0.8942
						**Genotype**	0.066	0.7983
						**Diet X genotype**	0.345	0.5608
	Female	0.06 ± 0.007	0.04 ± 0.003	0.04 ± 0.003	0.04 ± 0.003	**Diet**	3.042	0.0899
						**Genotype**	2.785	0.1041
						**Diet X genotype**	2.337	0.1354
Tetrdecanoyl-carnitine (C14)	Male	0.06 ± 0.009	0.06 ± 0.005	0.05 ± 0.004	0.06 ± 0.005	**Diet**	2.330	0.1356
						**Genotype**	1.388	0.2465
						**Diet X genotype**	0.377	0.5431
	Female	0.06 ± 0.008	0.05 ± 0.006	0.05 ± 0.004	0.03 ± 0.003	**Diet**	10.96	**0.0022**
						**Genotype**	5.097	**0.0303**
						**Diet X genotype**	0.125	0.7262
Tetradecenoyl-carnitine (C14:1)	Male	0.06 ± 0.008	0.06 ± 0.007	0.05 ± 0.006	0.05 ± 0.007	**Diet**	0.482	0.4920
						**Genotype**	0.130	0.7202
						**Diet X genotype**	0.308	0.5821
	Female	0.04 ± 0.01	0.04 ± 0.01	0.05 ± 0.004	0.04 ± 0.006	**Diet**	0.498	0.4850
						**Genotype**	3.628	0.0651
						**Diet X genotype**	0.012	0.9140
Hydroxytetra-decenoylcarnitine (14:1-OH)	Male	0.02 ± 0.002	0.02 ± 0.003	0.01 ± 0.003	0.02 ± 0.003	**Diet**	0.038	0.8461
						**Genotype**	4.780	**0.0354**
						**Diet X genotype**	1.418	0.2415
	Female	0.02 ± 0.003	0.02 ± 0.004	0.01 ± 0.002	0.02 ± 0.003	**Diet**	2.981	0.0931
						**Genotype**	0.526	0.4732
						**Diet X genotype**	1.161	0.2886
Tetradecadienyl-carnitine (14:2)	Male	0.02 ± 0.004	0.02 ± 0.003	0.02 ± 0.003	0.01 ± 0.003	**Diet**	1.412	0.2425
						**Genotype**	1.412	0.2425

						**Diet X genotype**	0.016	0.9021
	Female	0.02 ± 0.002	0.02 ± 0.003	0.01 ± 0.002	0.01 ± 0.002	**Diet**	0.886	0.3531
						**Genotype**	0.065	0.8002
						**Diet X genotype**	0.007	0.9340
Hydroxytetra-decadienylcarnitine (C14:2-OH)	Male	0.02 ± 0.003	0.02 ± 0.003	0.02 ± 0.003	0.02 ± 0.003	**Diet**	0.138	0.7124
						**Genotype**	0.848	0.3632
						**Diet X genotype**	0.357	0.5537
	Female	0.01 ± 0.003	0.02 ± 0.004	0.02 ± 0.003	0.02 ± 0.003	**Diet**	0.315	0.5781
						**Genotype**	1.571	0.2184
						**Diet X genotype**	0.016	0.8997
Hexadecanoyl-carnitine (C16)	Male	0.17 ± 0.02	0.15 ± 0.02	0.14 ± 0.009	0.18 ± 0.01	**Diet**	5.062 e-005	0.9944
						**Genotype**	0.869	0.3575
						**Diet X genotype**	4.678	**0.0373**
	Female	0.14 ± 0.02	0.13 ± 0.01	0.13 ± 0.009	0.11 ± 0.009	**Diet**	1.781	0.1907
						**Genotype**	2.058	0.1603
						**Diet X genotype**	0.141	0.7097
Hydroxyhexa-decanoylcarnitine (C16-OH)	Male	0.03 ± 0.006	0.03 ± 0.005	0.03 ± 0.002	0.03 ± 0.005	**Diet**	0.231	0.6335
						**Genotype**	0.110	0.7423
						**Diet X genotype**	0.252	0.6185
	Female	0.03 ± 0.003	0.02 ± 0.003	0.03 ± 0.003	0.02 ± 0.004	**Diet**	0.212	0.6478
						**Genotype**	3.397	0.0738
						**Diet X genotype**	0.518	0.4763
Hexadecenoyl-carnitine (C16:1)	Male	0.05 ± 0.005	0.05 ± 0.007	0.05 ± 0.006	0.04 ± 0.006	**Diet**	3.003	0.0917
						**Genotype**	0.140	0.7108
						**Diet X genotype**	0.693	0.4107
	Female	0.06 ± 0.007	0.05 ± 0.006	0.04 ± 0.003	0.03 ± 0.007	**Diet**	6.603	**0.0146**
						**Genotype**	1.522	0.2255
						**Diet X genotype**	0.757	0.3902
Hydroxyhexa-decenoylcarnitine (C16:1-OH)	Male	0.03 ± 0.003	0.02 ± 0.003	0.02 ± 0.002	0.01 ± 0.003	**Diet**	4.499	0.0409
						**Genotype**	3.738	0.0611
						**Diet X genotype**	0.592	0.4466
	Female	0.02 ± 0.002	0.01 ± 0.002	0.01 ± 0.002	0.02 ± 0.003	**Diet**	1.085	0.3048
						**Genotype**	0.097	0.7577

						**Diet X genotype**	14.51	**0.0005**
Hexadeacdienyl-carnitine (C16:2)	Male	0.02 ± 0.003	0.02 ± 0.002	0.02 ± 0.005	0.02 ± 0.003	**Diet**	1.022	0.3189
						**Genotype**	1.856	0.1816
						**Diet X genotype**	1.022	0.3189
	Female	0.02 ± 0.002	0.02 ± 0.003	0.02 ± 0.003	0.02 ± 0.003	**Diet**	4.205 e-006	0.9984
						**Genotype**	0.109	0.7433
						**Diet X genotype**	0.167	0.6857
Hydroxyhexa-decadienylcarnitine (C16:2-OH)	Male	0.03 ± 0.003	0.02 ± 0.002	0.03 ± 0.005	0.02 ± 0.003	**Diet**	0.577	0.8115
						**Genotype**	0.657	0.4228
						**Diet X genotype**	0.004	0.9524
	Female	0.02 ± 0.003	0.02 ± 0.002	0.02 ± 0.004	0.03 ± 0.003	**Diet**	0.959	0.3342
						**Genotype**	0.420	0.5213
						**Diet X genotype**	1.243	0.2725
Octadecanylcarnitine (C18)	Male	0.04 ± 0.005	0.04 ± 0.002	0.1 ± 0.01	0.1 ± 0.01	**Diet**	63.92	**<0.0001**
						**Genotype**	1.272	0.2669
						**Diet X genotype**	0.905	0.3478
	Female	0.05 ± 0.005	0.04 ± 0.004	0.07 ± 0.007	0.07 ± 0.006	**Diet**	20.29	**<0.0001**
						**Genotype**	0.443	0.5100
						**Diet X genotype**	0.071	0.7912
Octadecenoyl-carnitine (C18:1)	Male	0.1 ± 0.01	0.1 ± 0.008	0.2 ± 0.01	0.15 ± 0.01	**Diet**	12.75	**0.0010**
						**Genotype**	0.575	0.4534
						**Diet X genotype**	0.890	0.3518
	Female	0.1 ± 0.01	0.1 ± 0.02	0.14 ± 0.01	0.12 ± 0.008	**Diet**	5.322	**0.0271**
						**Genotype**	0.920	0.3441
						**Diet X genotype**	0.027	0.8644
Hydroxyocta-decenoylcarnitine (C18:1-OH)	Male	0.02 ± 0.003	0.02 ± 0.003	0.02 ± 0.003	0.02 ± 0.003	**Diet**	0.873	0.3563
						**Genotype**	0.013	0.9093
						**Diet X genotype**	0.293	0.5918
	Female	0.03 ± 0.003	0.02 ± 0.003	0.02 ± 0.003	0.02 ± 0.004	**Diet**	0.501	0.4836
						**Genotype**	0.025	0.8765
						**Diet X genotype**	0.036	0.8515
Octadecadienyl-carnitine (C18:2)	Male	0.04 ± 0.007	0.04 ± 0.005	0.03 ± 0.002	0.03 ± 0.004	**Diet**	7.872	**0.0081**
						**Genotype**	0.460	0.5021

						**Diet X genotype**	0.612	0.4392
	Female	0.05 ± 0.006	0.04 ± 0.005	0.03 ± 0.003	0.02 ± 0.002	**Diet**	24.26	**<0.0001**
						**Genotype**	3.216	0.0816
						**Diet X genotype**	0.712	0.4045
**Amino acids**								
Alanine (Ala)	Male	334.6 ± 33.02	318.6 ± 24.1	358.8 ± 29.404	429.7 ± 30.5	**Diet**	5.331	**0.0270**
						**Genotype**	0.879	0.3550
						**Diet X genotype**	2.195	0.1474
	Female	268.7 ± 15.8	262.8 ± 7.1	334.2 ± 30.6	423.4 ± 31.8	**Diet**	21.61	**<0.0001**
						**Genotype**	2.931	0.0957
						**Diet X genotype**	3.824	0.0585
Arginine (Arg)	Male	92.3 ± 18.6	118.2 ± 15.5	100.4 ± 11.0	170.6 ± 23.0	**Diet**	3.292	0.0796
						**Genotype**	8.284	**0.0073**
						**Diet X genotype**	1.756	0.1952
	Female	86.9 ± 8.1	106.5 ± 8.7	68.1 ± 3.2	83.6 ± 5.1	**Diet**	10.03	0.0032
						**Genotype**	7.111	**0.0115**
						**Diet X genotype**	0.102	0.7520
Aspargine (Asn)	Male	116.4 ± 13.6	107.3 ± 10.5	129.8 ± 7.7	168.1 ± 16.7	**Diet**	8.731	**0.0056**
						**Genotype**	1.346	0.2538
						**Diet X genotype**	3.553	0.0678
	Female	101.0 ± 7.4	113.1 ± 6.8	106.7 ± 7.0	118.0 ± 11.5	**Diet**	0.393	0.5347
						**Genotype**	1.927	0.1738
						**Diet X genotype**	0.002	0.9630
Aspartic acid (Asp)	Male	82.1 ± 7.6	88.5 ± 10.7	75.1 ± 5.8	96.6 ± 11.8	**Diet**	0.004	0.9503
						**Genotype**	2.269	0.1412
						**Diet X genotype**	0.665	0.4205
	Female	66.7 ± 6.7	61.0 ± 3.8	74.8 ± 7.4	104.7 ± 15.6	**Diet**	6.609	**0.0148**
						**Genotype**	1.459	0.2356
						**Diet X genotype**	3.123	0.0864
Citruline (Cit)	Male	42.2 ± 3.7	47.2 ± 3.9	44.5 ± 6.0	51.5 ± 5.5	**Diet**	0.446	0.5085
						**Genotype**	1.472	0.2332
						**Diet X genotype**	0.043	0.8367
	Female	53.6 ± 3.9	56.0 ± 4.1	45.4 ± 4.1	53.0 ± 3.1	**Diet**	2.165	0.1501
						**Genotype**	1.721	0.1981

						**Diet X genotype**	0.460	0.5023
Glutamine (Gln)	Male	608.8 ± 32.9	525.4 ± 21.3	494 ± 17.057	585.7 ± 28.7	**Diet**	1.152	0.2904
						**Genotype**	0.027	0.8707
						**Diet X genotype**	11.90	**0.0015**
	Female	549.4 ± 27.7	507.6 ± 29.8	581.1 ± 34.1	541.4 ± 22.5	**Diet**	1.293	0.2633
						**Genotype**	2.001	0.1660
						**Diet X genotype**	0.001	0.9705
Glutamic acid (Glu)	Male	162.7 ± 37.2	219.3 ± 36.8	109.5 ± 26.8	113.1 ± 22.9	**Diet**	6.638	**0.0148**
						**Genotype**	0.946	0.3381
						**Diet X genotype**	0.736	0.3974
	Female	114.5 ± 35.2	41.8 ± 19.6	88.5 ± 29.3	36.8 ± 11.0	**Diet**	0.350	0.5580
						**Genotype**	5.600	**0.0238**
						**Diet X genotype**	0.161	0.6906
Glycine (Gly)	Male	273.2 ± 13.9	221.7 ± 10.6	193.5 ± 10.4	256 ± 16.8	**Diet**	2.907	0.0973
						**Genotype**	0.169	0.6836
						**Diet X genotype**	18.36	**0.0001**
	Female	210.4 ± 11.4	179.2 ± 5.5	181.8 ± 11.7	197 ± 11.2	**Diet**	0.265	0.6099
						**Genotype**	0.578	0.4523
						**Diet X genotype**	4.868	**0.0340**
Histidine (His)	Male	63.5 ± 5.4	56.3 ± 2.2	66.7 ± 2.9	85.1 ± 8.0	**Diet**	9.230	**0.0046**
						**Genotype**	1.139	0.2934
						**Diet X genotype**	5.916	**0.0204**
	Female	60.7 ± 3.8	61.2 ± 1.8	69.2 ± 4.1	72.8 ± 3.8	**Diet**	7.876	**0.0081**
						**Genotype**	0.325	0.5722
						**Diet X genotype**	0.189	0.6667
Isoleucine (Ile)	Male	84.0 ± 6.4	98.9 ± 8.7	115.1 ± 9.0	131.0 ± 12.8	**Diet**	11.08	**0.0020**
						**Genotype**	2.634	0.1133
						**Diet X genotype**	0.003	0.9574
	Female	86.1 ± 6.6	96.4 ± 5.4	82.0 ± 5.0	94.2 ± 13.6	**Diet**	0.135	0.7160
						**Genotype**	1.716	0.1988
						**Diet X genotype**	0.013	0.9098
Leucine (Leu)	Male	150.9 ± 22.8	160.1 ± 21.7	194 ± 12.4	237.6 ± 26.8	**Diet**	8.005	**0.0077**
						**Genotype**	1.532	0.2241
						**Diet X genotype**	0.652	0.4250

	Female	127.07 ± 12.3	138.7 ± 7.6	129.5 ± 8.5	152.7 ± 19.8	**Diet**<br>**Genotype**<br>**Diet X genotype**	0.386<br>1.734<br>0.194	0.5384<br>0.1965<br>0.6628
Lysine (Lys)	Male	296.3 ± 59.0	312.1 ± 62.4	364.7 ± 40.5	526.2 ± 75.3	**Diet**<br>**Genotype**<br>**Diet X genotype**	5.571<br>2.194<br>1.483	**0.0240**<br>0.1475<br>0.2314
	Female	233.3 ± 12.6	237.7 ± 9.4	322.2 ± 28.6	348.7 ± 27.1	**Diet**<br>**Genotype**<br>**Diet X genotype**	21.18<br>0.505<br>0.260	**<0.0001**<br>0.4821<br>0.6137
Methionine (Met)	Male	80.6 ± 11.7	73.5 ± 9.3	87.9 ± 8.1	119.2 ± 14.8	**Diet**<br>**Genotype**<br>**Diet X genotype**	5.532<br>1.158<br>2.915	**0.0243**<br>0.2891<br>0.0964
	Female	69.5 ± 7.1	72.8 ± 3.4	70.5 ± 6.1	89.1 ± 7.0	**Diet**<br>**Genotype**<br>**Diet X genotype**	1.941<br>3.112<br>1.510	0.1724<br>0.0865<br>0.2273
Ornithine (Orn)	Male	55.1 ± 4.7	51.4 ± 4.3	41.6 ± 3.9	51.4 ± 3.9	**Diet**<br>**Genotype**<br>**Diet X genotype**	2.590<br>0.520<br>2.559	0.1163<br>0.4754<br>0.1184
	Female	35.5 ± 3.0	34.4 ± 2.6	41.8 ± 7.2	47.9 ± 8.9	**Diet**<br>**Genotype**<br>**Diet X genotype**	2.534<br>0.165<br>0.337	0.1204<br>0.6868<br>0.5653
Phenylalanine (Phe)	Male	61.3 ± 5.6	63.7 ± 1.7	61.0 ± 2.6	90.5 ± 14.2	**Diet**<br>**Genotype**<br>**Diet X genotype**	2.774<br>4.022<br>2.896	0.1066<br>0.0527<br>0.0977
	Female	61.3 ± 5.6	63.7 ± 1.7	61.0 ± 2.6	65.9 ± 4.7	**Diet**<br>**Genotype**<br>**Diet X genotype**	0.048<br>0.821<br>0.095	0.8271<br>0.3710<br>0.7594
Proline (Pro)	Male	102.6 ± 15.3	90.7 ± 10.3	140.5 ± 16.6	172.4 ± 20.5	**Diet**<br>**Genotype**<br>**Diet X genotype**	13.84<br>0.387<br>1.858	**0.0007**<br>0.5376<br>0.1814
	Female	82.5 ± 11.1	89.3 ± 5.8	100.3 ± 11.2	129.5 ± 18.0	**Diet**<br>**Genotype**<br>**Diet X genotype**	5.334<br>2.040<br>0.798	**0.0269**<br>0.1621<br>0.3779
Serine (Ser)	Male	133.3 ± 19.04	130.0± 19.1	130.8 ± 7.3	201.7 ± 21.0	**Diet**	3.923	0.0553

						**Genotype**	3.739	0.0610
						**Diet X genotype**	4.510	**0.0406**
	Female	128.5 ± 5.6	128.7 ± 6.6	142.1 ± 8.2	155.8 ± 11.7	**Diet**	5.769	**0.0218**
						**Genotype**	0.679	0.4156
						**Diet X genotype**	0.632	0.4320
Threonine (Thr)	Male	145.9 ± 14.6	134.3 ± 14.4	204.1 ± 14.5	249.8 ± 24.6	**Diet**	24.31	**<0.0001**
						**Genotype**	0.933	0.3407
						**Diet X genotype**	2.653	0.1121
	Female	139.9 ± 7.4	150.4 ± 8.9	174.3 ± 22.1	216.1 ± 14.0	**Diet**	11.84	**0.0015**
						**Genotype**	3.236	0.0807
						**Diet X genotype**	1.153	0.2902
Tryptophane (Trp)	Male	79.7 ± 2.8	80.2 ± 6.5	58.7 ± 6.0	76.9 ± 9.9	**Diet**	3.052	0.0894
						**Genotype**	1.777	0.1911
						**Diet X genotype**	1.604	0.2137
	Female	103.2 ± 9.5	104.6 ± 6.0	75.8 ± 4.7	92.6 ± 6.4	**Diet**	7.995	**0.0077**
						**Genotype**	1.723	0.1979
						**Diet X genotype**	1.225	0.2759
Tyrosine (Tyr)	Male	73.8 ± 9.9	77.4 ± 7.8	100.1 ± 11.5	131.7 ± 19.2	**Diet**	9.600	**0.0038**
						**Genotype**	1.831	0.1847
						**Diet X genotype**	1.160	0.2888
	Female	72.0 ± 9.8	76.8 ± 3.5	82.7 ± 7.3	104.8 ± 8.5	**Diet**	6.172	**0.0179**
						**Genotype**	2.955	0.0944
						**Diet X genotype**	1.217	0.2774
Valine (Val)	Male	202.7 ± 10.5	217.3 ± 16.5	299.4 ± 18.6	321.1 ± 32.7	**Diet**	21.39	**<0.0001**
						**Genotype**	0.702	0.4077
						**Diet X genotype**	0.027	0.8715
	Female	196.4 ± 15.8	215.4± 12.3	199.4 ± 14.3	238.3 ± 23.9	**Diet**	0.554	0.4615
						**Genotype**	2.784	0.1041
						**Diet X genotype**	0.327	0.5711
**Biogenic Amines**								
Dopamine	Male	1.8 ± 0.3	1.6 ± 0.16	2.5 ± 0.4	3.3 ± 0.6	**Diet**	8.301	**0.0068**
						**Genotype**	0.471	0.4971
						**Diet X genotype**	1.913	0.1756
	Female	1.7 ± 0.2	3.1 ± 0.9	3.8± 0.7	3.1 ± 0.7	**Diet**	2.160	0.1517

						**Genotype**<br>**Diet X genotype**	0.224<br>2.118	0.6394<br>0.1556
Carnosine	Male	3.1 ± 0.4	2.6 ± 0.2	1.7 ± 0.2	1.8 ± 0.2	**Diet**<br>**Genotype**<br>**Diet X genotype**	18.14<br>0.879<br>1.368	**0.0002**<br>0.3550<br>0.2503
	Female	1.3 ± 0.1	1.2 ± 0.1	1.4 ± 0.1	1.1 ± 0.07	**Diet**<br>**Genotype**<br>**Diet X genotype**	0.0001<br>1.386<br>0.523	0.9920<br>0.2486<br>0.4752
Creatine	Male	5.2 ± 0.6	4.4 ± 0.5	3.2 ± 0.5	3.8 ± 0.3	**Diet**<br>**Genotype**<br>**Diet X genotype**	7.041<br>0.045<br>1.764	**0.0119**<br>0.8329<br>0.1927
	Female	4.7 ± 0.5	3.8 ± 0.4	4.6 ± 0.4	5.5 ± 0.3	**Diet**<br>**Genotype**<br>**Diet X genotype**	4.016<br>0.026<br>5.055	0.0529<br>0.8725<br>0.0310
Histamine	Male	1.1 ± 0.1	0.9 ± 0.05	0.8 ± 0.04	0.9 ± 0.09	**Diet**<br>**Genotype**<br>**Diet X genotype**	3.796<br>0.079<br>6.719	0.0594<br>0.7798<br>**0.0138**
	Female	1.6 ± 0.1	1.4 ± 0.2	1.4 ± 0.1	1.2 ± 0.07	**Diet**<br>**Genotype**<br>**Diet X genotype**	3.644<br>2.473<br>0.008	0.0645<br>0.1248<br>0.9286
Putrescine	Male	0.5 ± 0.09	0.5 ± 0.06	0.6 ± 0.07	0.7 ± 0.1	**Diet**<br>**Genotype**<br>**Diet X genotype**	3.205<br>1.599<br>0.129	0.0821<br>0.2144<br>0.7221
	Female	0.4 ± 0.05	0.4 ± 0.04	0.5 ± 0.05	0.4 ± 0.03	**Diet**<br>**Genotype**<br>**Diet X genotype**	0.993<br>1.045<br>0.140	0.3258<br>0.3137<br>0.7108
Spermidine	Male	0.03 ± 0.002	0.02 ± 0.001	0.03 ± 0.002	0.04 ± 0.005	**Diet**<br>**Genotype**<br>**Diet X genotype**	7.041<br>5.588<br>8.286	**0.0120**<br>**0.0239**<br>**0.0069**
	Female	0.03 ± 0.003	0.03 ± 0.004	0.03 ± 0.002	0.03 ± 0.003	**Diet**<br>**Genotype**<br>**Diet X genotype**	0.149<br>0.006<br>1.448	0.7019<br>0.9371<br>0.2371
Spermine	Male	0.6 ± 0.07	0.5 ± 0.03	0.6 ± 0.04	0.8 ± 0.06	**Diet**<br>**Genotype**	6.525<br>0.151	**0.0151**<br>0.7002

						**Diet X genotype**	8.099	**0.0074**
	Female	0.6 ± 0.04	0.5 ± 0.05	0.6 ± 0.06	0.6 ± 0.05	**Diet**	0.166	0.6859
						**Genotype**	0.176	0.6772
						**Diet X genotype**	0.661	0.4217
Trans-4-Hydroxyproline (t4-OH-Pro)	Male	11.3 ± 0.9	9.6 ± 0.9	11.5 ± 1.6	10.7 ± 1.3	**Diet**	0.305	0.5844
						**Genotype**	1.213	0.2782
						**Diet X genotype**	0.138	0.7123
	Female	6.3 ± 0.4	5.8 ± 0.3	5.04 ± 0.4	5.2 ± 0.4	**Diet**	5.380	**0.0265**
						**Genotype**	0.154	0.6976
						**Diet X genotype**	0.710	0.4053
Taurine	Male	444.7 ± 26.2	403.2 ± 17.0	370.3 ± 16.5	428.6 ± 18.8	**Diet**	1.498	0.2289
						**Genotype**	0.176	0.6772
						**Diet X genotype**	6.215	**0.0174**
	Female	339.2 ± 15.4	303.2 ± 11.8	357.8 ± 11.0	349.1 ± 15.6	**Diet**	5.512	**0.0247**
						**Genotype**	2.647	0.1127
						**Diet X genotype**	0.987	0.3274
Kynurenine	Male	1.2 ± 0.1	1.02 ± 0.1	0.7 ± 0.09	1.02 ± 0.1	**Diet**	4.638	**0.0392**
						**Genotype**	0.729	0.3997
						**Diet X genotype**	4.465	**0.0427**
	Female	1.6 ± 0.09	1.3 ± 0.1	1.03 ± 0.1	0.9 ± 0.1	**Diet**	19.11	**0.0001**
						**Genotype**	4.088	0.0514
						**Diet X genotype**	0.350	0.5582
**Glycerophospholipids**								
Lysophosphatidylcholine acyls								
lysoPC a C14:0	Male	18.8 ± 1.2	22.2 ± 0.5	30.04 ± 1.8	31.01 ± 1.5	**Diet**	55.79	**<0.0001**
						**Genotype**	2.664	0.1114
						**Diet X genotype**	0.830	0.3684
	Female	14.8 ± 0.3	17.3 ± 0.5	30.0 ± 2.4	33.1 ± 2.2	**Diet**	83.00	**<0.0001**
						**Genotype**	2.705	0.1090
						**Diet X genotype**	0.040	0.8420
lysoPC a C16:0	Male	745.8 ± 86.8	779.1 ± 18.1	806.1 ± 105.0	751 ± 31.5	**Diet**	0.052	0.8207
						**Genotype**	0.024	0.8779
						**Diet X genotype**	0.393	0.5347
	Female	487.8 ± 15.6	500.3± 14.6	584.5 ± 25.6	691.3 ± 47.4	**Diet**	23.64	**<0.0001**

						**Genotype**	4.067	0.0514
						**Diet X genotype**	2.538	0.1201
lysoPC a C16:1	Male	40.5 ± 11.9	25.9 ± 1.8	15.7 ± 3.1	12.3 ± 1.3	**Diet**	9.448	**0.0040**
						**Genotype**	2.070	0.1589
						**Diet X genotype**	0.807	0.3751
	Female	11.1 ± 1.05	11.6 ± 0.9	13.2 ± 1.2	14.0 ± 1.1	**Diet**	4.442	**0.0423**
						**Genotype**	0.324	0.5726
						**Diet X genotype**	0.012	0.9124
lysoPC a C17:0	Male	13.3 ± 1.1	11.7 ± 0.7	14.3 ± 2.0	13.8 ±1.3	**Dite**	1.403	0.2441
						**Genotype**	0.567	0.4570
						**Diet X genotype**	0.183	0.6737
	Female	10.6 ± 0.9	10.9 ± 0.9	10.3 ± 0.6	12.2 ± 0.9	**Diet**	0.359	0.5527
						**Genotype**	1.664	0.2055
						**Diet X genotype**	1.014	0.3209
lysoPC a C18:0	Male	67.1 ± 6.1	70.4 ± 2.2	124.4 ± 15.5	135.6 ± 13.5	**Diet**	32.16	**<0.0001**
						**Genotype**	0.444	0.5094
						**Diet X genotype**	0.133	0.7176
	Female	73.6 ± 4.6	74.9 ± 4.8	108.9 ± 5.8	131.1 ± 12.1	**Diet**	36.15	**<0.0001**
						**Genotype**	2.385	0.1315
						**Diet X genotype**	1.885	0.1785
lysoPC a C18:1	Male	210.4 ± 19.5	203.2 ± 10.3	312.2 ± 42.4	283.7 ± 15.5	**Diet**	13.17	**0.0009**
						**Genotype**	0.505	0.4818
						**Diet X genotype**	0.180	0.6740
	Female	130.4 ± 11.4	142.9± 13.1	268.9 ± 22.2	311.2 ± 32.6	**Diet**	48.73	**<0.0001**
						**Genotype**	1.555	0.2207
						**Diet X genotype**	0.460	0.5022
lysoPC a C18:2	Male	393 ± 44.7	444.3 ± 30.0	693.7 ± 78.4	650 ± 49.1	**Diet**	21.93	**<0.0001**
						**Genotype**	0.005	0.9444
						**Diet X genotype**	0.772	0.3857
	Female	288.2 ± 16.3	333.8 ± 24.4	561.5 ± 46.5	603.3 ± 64.8	**Diet**	39.24	**<0.0001**
						**Genotype**	1.017	0.3202
						**Diet X genotype**	0.002	0.9655
lysoPC a C20:3	Male	43.6 ± 6.9	40.2 ± 3.1	57.4 ± 8.7	54.6 ± 4.6	**Diet**	5.165	**0.0293**
						**Genotype**	0.254	0.6177

						**Diet X genotype**	0.003	0.9570
	Female	21.7 ± 1.8	23.6 ± 2.6	52.6 ± 4.3	54.5 ± 4.8	**Diet**	71.97	**<0.0001**
						**Genotype**	0.273	0.6046
						**Diet X genotype**	0.0003	0.9866
lysoPC a C20:4	Male	108.6 ± 15.6	110.9 ± 7.1	219.1 ± 35.3	179 ± 12.7	**Diet**	18.72	**0.0001**
						**Genotype**	0.843	0.3647
						**Diet X genotype**	1.053	0.3117
	Female	131.05 ± 6.3	118.3± 9.6	292 ± 20.0	313.4 ± 35.6	**Diet**	67.45	**<0.0001**
						**Genotype**	0.040	0.8436
						**Diet X genotype**	0.622	0.4357
lysoPC a C24:0	Male	3.2 ± 0.3	3.5 ± 0.2	5.05 ± 0.5	6.5 ± 0.9	**Diet**	21.08	**<0.0001**
						**Genotype**	2.787	0.1037
						**Diet X genotype**	1.192	0.2823
	Female	4.2 ± 0.6	2.8 ± 0.3	4.9 ± 0.4	5.2 ± 0.4	**Diet**	13.27	**0.0009**
						**Genotype**	1.724	0.1977
						**Diet X genotype**	4.178	**0.0485**
lysoPC a C26:0	Male	2.7 ± 0.5	2.6 ± 0.3	4.0 ± 0.5	5.5 ± 1.0	**Diet**	10.48	**0.0026**
						**Genotype**	1.234	0.2739
						**Diet X genotype**	1.600	0.2140
	Female	3.3 ± 0.5	1.8 ± 0.3	3.4 ± 0.4	3.4 ± 0.5	**Diet**	3.686	0.0630
						**Genotype**	3.153	0.0845
						**Diet X genotype**	3.324	0.0768
lysoPC a C26:1	Male	1.0 ± 0.2	0.9 ± 0.09	1.4 ± 0.1	1.8 ± 0.3	**Diet**	12.66	**0.0011**
						**Genotype**	1.225	0.2758
						**Diet X genotype**	2.246	0.1427
	Female	1.5 ± 0.2	0.7 ± 0.1	1.5 ± 0.17	1.6 ± 0.3	**Diet**	4.152	**0.0492**
						**Genotype**	3.358	0.0754
						**Diet X genotype**	4.722	**0.0366**
lysoPC a C28:0	Male	1.3 ± 0.1	1.1 ± 0.1	2.2 ± 0.3	3.1 ± 0.6	**Diet**	19.83	**<0.0001**
						**Genotype**	1.090	0.3034
						**Diet X genotype**	2.829	0.1012
	Female	2.0 ± 0.3	1.0 ± 0.1	2.0 ± 0.2	2.3 ± 0.3	**Diet**	6.531	**0.0151**
						**Genotype**	1.354	0.2525
						**Diet X genotype**	6.138	**0.0182**

lysoPC a C28:1	Male	1.0 ± 0.17	1.04 ± 0.1	1.7 ± 0.2	2.2 ± 0.3	**Diet**<br>**Genotype**<br>**Diet X genotype**	16.96<br>1.409<br>0.773	**0.0002**<br>0.2431<br>0.3851
	Female	1.5 ± 0.2	0.7 ± 0.1	1.7 ± 0.2	1.7 ± 0.2	**Diet**<br>**Genotype**<br>**Diet X genotype**	7.463<br>3.802<br>2.747	**0.0098**<br>0.0592<br>0.1064
Phosphatidylcholine diacyls								
PC aa C24:0	Male	0.2 ± 0.05	0.2 ± 0.03	0.3 ± 0.04	0.4 ± 0.06	**Diet**<br>**Genotype**<br>**Diet X genotype**	10.72<br>0.010<br>0.978	**0.0024**<br>0.9209<br>0.3293
	Female	0.5 ± 0.1	0.2 ± 0.04	0.5 ± 0.06	0.5 ± 0.05	**Diet**<br>**Genotype**<br>**Diet X genotype**	6.823<br>6.983<br>1.695	**0.0132**<br>**0.0122**<br>0.2015
PC aa C26:0	Male	1.9 ± 0.5	1.9 ± 0.2	2.4 ± 0.2	2.9 ± 0.6	**Diet**<br>**Genotype**<br>**Diet X genotype**	3.323<br>0.258<br>0.698	0.0769<br>0.6148<br>0.4093
	Female	2.3 ± 0.3	1.3 ± 0.2	2.07 ± 0.3	2.05 ± 0.23	**Diet**<br>**Genotype**<br>**Diet X genotype**	0.987<br>3.517<br>3.198	0.3274<br>0.0691<br>0.0824
PC aa C28:1	Male	0.5 ± 0.09	0.4 ± 0.04	0.7 ± 0.07	0.9 ± 0.1	**Diet**<br>**Genotype**<br>**Diet X genotype**	21.58<br>0.821<br>1.937	**<0.0001**<br>0.3712<br>0.1727
	Female	0.6 ± 0.07	0.3 ± 0.04	0.8 ± 0.07	0.8 ± 0.09	**Diet**<br>**Genotype**<br>**Diet X genotype**	22.99<br>3.961<br>2.069	**<0.0001**<br>0.0544<br>0.1592
PC aa C30:0	Male	1.6 ± 0.5	1.3 ± 0.2	1.1 ± 0.1	1.8 ± 0.2	**Diet**<br>**Genotype**<br>**Diet X genotype**	0.0002<br>0.342<br>2.463	0.9902<br>0.5622<br>0.1253
	Female	1.0 ± 0.1	1.0 ± 0.1	1.6 ± 0.2	1.8 ± 0.3	**Diet**<br>**Genotype**<br>**Diet X genotype**	12.58<br>0.140<br>0.077	**0.0011**<br>0.7103<br>0.7825
PC aa C30:2	Male	0.2 ± 0.07	0.1 ± 0.03	0.2 ± 0.04	0.2 ± 0.05	**Diet**<br>**Genotype**<br>**Diet X genotype**	0.250<br>0.104<br>2.179	0.6208<br>0.7491<br>0.1500

	Female	0.1 ± 0.02	0.1 ± 0.04	0.2 ± 0.03	0.2 ± 0.03	**Diet**<br>**Genotype**<br>**Diet X genotype**	5.992<br>0.096<br>1.152	**0.0207**<br>0.7591<br>0.2919
PC aa C32:0	Male	17.02 ± 1.3	18.8 ± 2.1	14.8 ± 1.4	23.4 ± 2.8	**Diet**<br>**Genotype**<br>**Diet X genotype**	0.365<br>6.485<br>2.866	0.5495<br>**0.0154**<br>0.0994
	Female	16.3 ± 1.6	17.4 ± 1.7	21.7 ± 2.6	23.7 ± 2.9	**Diet**<br>**Genotype**<br>**Diet X genotype**	6.384<br>0.420<br>0.039	**0.0162**<br>0.5213<br>0.8443
PC aa C32:1	Male	11.04 ± 1.2	12.4 ± 1.5	7.6 ± 1.0	10.04 ± 1.2	**Dite**<br>**Genotype**<br>**Diet X genotype**	5.506<br>2.378<br>0.184	**0.0247**<br>0.1320<br>0.6710
	Female	5.2 ± 0.7	5.7 ± 0.8	7.5 ± 1.2	8.3 ± 1.7	**Diet**<br>**Genotype**<br>**Diet X genotype**	4.025<br>0.319<br>0.017	0.0526<br>0.5757<br>0.8970
PC aa C32:2	Male	1.4 ± 0.09	1.4 ± 0.1	0.9 ± 0.1	0.9 ± 0.07	**Diet**<br>**Genotype**<br>**Diet X genotype**	20.05<br>0.023<br>0.008	**<0.0001**<br>0.8807<br>0.9285
	Female	0.6 ± 0.05	0.6 ± 0.05	0.8 ± 0.07	0.7 ± 0.07	**Diet**<br>**Genotype**<br>**Diet X genotype**	7.650<br>0.338<br>2.289	**0.0090**<br>0.5645<br>0.1392
PC aa C32:3	Male	0.17 ± 0.02	0.14 ± 0.01	0.17 ± 0.02	0.2 ± 0.02	**Diet**<br>**Genotype**<br>**Diet X genotype**	4.917<br>0.354<br>5.007	**0.0332**<br>0.5557<br>**0.0317**
	Female	0.15 ± 0.02	0.13 ± 0.01	0.2 ± 0.02	0.19 ± 0.02	**Diet**<br>**Genotype**<br>**Diet X genotype**	8.464<br>0.906<br>0.423	**0.0063**<br>0.3478<br>0.5197
PC aa C34:1	Male	206.6 ± 8.0	219.4 ± 15.5	259.1 ± 19.4	277.7 ± 7.5	**Diet**<br>**Genotype**<br>**Diet X genotype**	16.01<br>1.288<br>0.043	**0.0003**<br>0.2641<br>0.8366
	Female	83 ± 8.2	105.7 ± 7.8	175.7 ± 12.8	187.6 ± 12.0	**Diet**<br>**Genotype**<br>**Diet X genotype**	68.10<br>2.668<br>0.259	**<0.0001**<br>0.1113<br>0.6141

PC aa C34:2	Male	339 ± 14.7	404.6 ± 24.3	445.1 ± 28.018	513.5 ± 26.2	**Diet**	19.55	**<0.0001**
						**Genotype**	7.594	**0.0092**
						**Diet X genotype**	0.003	0.9544
	Female	171.4 ± 14.5	204.3 ± 8.02	288.9 ± 30.5	278.8 ± 16.4	**Diet**	23.87	**<0.0001**
						**Genotype**	0.338	0.5648
						**Diet X genotype**	1.200	0.2809
PC aa C34:3	Male	12.7 ± 0.7	13.6 ± 0.9	10.5 ± 1.01	10.9 ± 0.5	**Diet**	9.956	**0.0032**
						**Genotype**	0.765	0.3875
						**Diet X genotype**	0.076	0.7839
	Female	4.8 ± 0.3	5.1 ± 0.3	6.6 ± 0.7	5.7 ± 0.4	**Diet**	5.933	**0.0201**
						**Genotype**	0.367	0.5488
						**Diet X genotype**	1.858	0.1815
PC aa C34:4	Male	0.4 ± 0.03	0.5 ± 0.05	0.5 ± 0.08	0.5 ± 0.03	**Diet**	1.063	0.3095
						**Genotype**	0.050	0.8241
						**Diet X genotype**	2.109	0.1554
	Female	0.2 ± 0.02	0.2 ± 0.03	0.4 ± 0.04	0.3 ± 0.03	**Diet**	20.24	**<0.0001**
						**Genotype**	0.968	0.3319
						**Diet X genotype**	0.056	0.8143
PC aa C36:0	Male	2.4 ± 0.1	2.6 ± 0.2	4.5 ± 0.3	5.0 ± 0.3	**Diet**	88.85	**<0.0001**
						**Genotype**	2.265	0.1413
						**Diet X genotype**	0.213	0.6472
	Female	2.04 ± 0.2	2.1 ± 0.1	4.7 ± 0.5	4.2 ± 0.4	**Diet**	45.44	**<0.0001**
						**Genotype**	0.338	0.5647
						**Diet X genotype**	0.667	0.4196
PC aa C36:1	Male	19.4 ± 1.04	21.4 ± 1.7	65.2 ± 3.8	75.7 ± 4.5	**Diet**	246.5	**<0.0001**
						**Genotype**	3.865	0.0573
						**Diet X genotype**	1.799	0.1885
	Female	13.5 ± 1.1	16.3 ± 1.7	46.3 ± 4.6	48.7 ± 3.8	**Diet**	102.2	**<0.0001**
						**Genotype**	0.663	0.4210
						**Diet X genotype**	0.003	0.9571
PC aa C36:2	Male	174 ± 9.5	206.8 ± 10.7	400.6 ± 23.9	474.5 ± 27.6	**Diet**	151.9	**<0.0001**
						**Genotype**	7.079	**0.0117**
						**Diet X genotype**	1.050	0.3125
	Female	133.04 ± 13.8	162.7 ± 13.9	300.2 ± 27.7	314.7 ± 30.0	**Diet**	47.86	**<0.0001**

						**Genotype**	0.916	0.3450
						**Diet X genotype**	0.108	0.7444
PC aa C36:3	Male	86.6 ± 6.8	90.7 ± 4.9	93.5 ± 6.8	106.4 ± 4.4	**Diet**	3.873	0.0570
						**Genotype**	2.165	0.1501
						**Diet X genotype**	0.570	0.4553
	Female	40.5 ± 4.2	50.5 ± 4.8	70.9 ± 6.4	71.1 ± 5.4	**Diet**	23.35	**<0.0001**
						**Genotype**	0.933	0.3407
						**Diet X genotype**	0.861	0.3598
PC aa C36:4	Male	124.8 ± 5.5	135.2 ± 5.7	181.7 ± 18.1	187.5 ± 6.6	**Diet**	26.24	**<0.0001**
						**Genotype**	0.575	0.4532
						**Diet X genotype**	0.046	0.8308
	Female	88.9 ± 7.1	91.1 ± 7.07	169.1 ± 12.6	172.1 ± 13.8	**Diet**	56.28	**<0.0001**
						**Genotype**	0.060	0.8076
						**Diet X genotype**	0.001	0.9732
PC aa C36:5	Male	7.5 ± 0.5	9.4 ± 1.0	5.6 ± 0.7	6.2 ± 0.4	**Diet**	12.81	**0.0010**
						**Genotype**	3.109	0.0866
						**Diet X genotype**	0.938	0.3394
	Female	3.8 ± 0.4	4.2 ± 0.3	4.8 ± 0.4	4.2 ± 0.4	**Diet**	1.830	0.1848
						**Genotype**	0.093	0.7617
						**Diet X genotype**	1.485	0.2312
PC aa C36:6	Male	0.3 ± 0.04	0.3 ± 0.03	0.3 ± 0.04	0.3 ± 0.02	**Diet**	0.527	0.4729
						**Genotype**	0.699	0.4088
						**Diet X genotype**	0.026	0.8730
	Female	0.13 ± 0.015	0.12 ± 0.03	0.2 ± 0.02	0.2 ± 0.03	**Diet**	12.56	**0.0011**
						**Genotype**	0.107	0.7461
						**Diet X genotype**	0.0003	0.9873
PC aa C38:0	Male	1.4 ± 0.1	1.5 ± 0.1	2.5 ± 0.19	3.3 ± 0.3	**Diet**	62.14	**<0.0001**
						**Genotype**	5.796	**0.0215**
						**Diet X genotype**	4.678	**0.0375**
	Female	1.3 ± 0.09	1.1 ± 0.1	2.8 ± 0.2	2.7 ± 0.2	**Diet**	82.96	**<0.0001**
						**Genotype**	0.456	0.5041
						**Diet X genotype**	0.062	0.8045
PC aa C38:1	Male	1.05 ± 0.17	1.02 ± 0.07	1.7 ± 0.2	2.5 ± 0.4	**Diet**	21.15	**<0.0001**
						**Genotype**	2.541	0.1199

						**Diet X genotype**	3.037	0.0902
	Female	0.9 ± 0.1	0.6 ± 0.1	1.5 ± 0.1	1.6 ± 0.1	**Diet**	38.17	**<0.0001**
						**Genotype**	0.578	0.4521
						**Diet X genotype**	2.429	0.1281
PC aa C38:3	Male	20.0 ± 1.5	21.9 ± 1.2	27.0 ± 2.2	30.8 ± 1.9	**Diet**	20.75	**<0.0001**
						**Genotype**	2.744	0.1066
						**Diet X genotype**	0.308	0.5824
	Female	12.3 ± 1.03	15.4 ± 1.5	23.6 ± 1.7	24.2 ± 1.9	**Diet**	40.60	**<0.0001**
						**Genotype**	1.391	0.2463
						**Diet X genotype**	0.657	0.4230
PC aa C38:4	Male	54.7 ± 4.2	58.7 ± 2.8	117.9 ± 12.3	120.3 ± 8.7	**Diet**	59.08	**<0.0001**
						**Genotype**	0.154	0.6973
						**Diet X genotype**	0.009	0.9235
	Female	72.9 ± 6.9	71.9 ± 7.04	145.3 ± 8.4	156.7 ± 17.9	**Diet**	48.89	**<0.0001**
						**Genotype**	0.216	0.6452
						**Diet X genotype**	0.304	0.5852
PC aa C38:5	Male	32.2 ± 2.06	31.3 ± 1.5	33.04 ± 3.9	32.0 ± 1.8	**Diet**	0.091	0.7648
						**Genotype**	0.145	0.7060
						**Diet X genotype**	0.001	0.9716
	Female	20.7 ± 1.7	21.3 ± 2.4	35.1 ± 2.4	35.08 ± 3.9	**Diet**	26.42	**<0.0001**
						**Genotype**	0.008	0.9280
						**Diet X genotype**	0.012	0.9136
PC aa C38:6	Male	86.0 ± 3.6	80.6 ± 3.07	95.3 ± 10.07	97.4 ± 5.3	**Diet**	4.304	**0.0454**
						**Genotype**	0.071	0.7917
						**Diet X genotype**	0.358	0.5535
	Female	49.1 ± 3.2	48.6 ± 4.6	92.2 ± 6.3	95.2 ± 7.09	**Diet**	65.30	**<0.0001**
						**Genotype**	0.049	0.8255
						**Diet X genotype**	0.101	0.7522
PC aa C40:2	Male	0.6 ± 0.03	0.6 ± 0.05	0.8 ± 0.05	0.9 ± 0.07	**Diet**	15.17	**0.0004**
						**Genotype**	3.131	0.0855
						**Diet X genotype**	2.085	0.1577
	Female	0.5 ± 0.05	0.4 ± 0.04	0.7 ± 0.06	0.7 ± 0.07	**Diet**	24.47	**<0.0001**
						**Genotype**	0.164	0.6879
						**Diet X genotype**	0.469	0.4978

PC aa C40:3	Male	1.03 ± 0.06	0.8 ± 0.06	0.9 ± 0.06	0.9 ± 0.05	**Diet**<br>**Genotype**<br>**Diet X genotype**	0.316<br>5.722<br>2.050	0.5777<br>**0.0223**<br>0.1610
	Female	0.4 ± 0.03	0.4 ± 0.04	0.6 ± 0.02	0.5 ± 0.04	**Diet**<br>**Genotype**<br>**Diet X genotype**	16.17<br>0.494<br>0.696	**0.0003**<br>0.4870<br>0.4097
PC aa C40:4	Male	2.02 ± 0.3	2.0 ± 0.2	3.2 ± 0.2	3.2 ± 0.2	**Diet**<br>**Genotype**<br>**Diet X genotype**	34.77<br>3.007e-005<br>0.053	**<0.0001**<br>0.9957<br>0.8195
	Female	1.04 ± 0.07	1.2 ± 0.1	1.6 ± 0.1	1.6 ± 0.1	**Diet**<br>**Genotype**<br>**Diet X genotype**	22.86<br>0.772<br>0.150	**<0.0001**<br>0.3857<br>0.7012
PC aa C40:5	Male	3.8 ± 0.5	4.02 ± 0.3	5.4 ± 0.5	4.8 ± 0.4	**Diet**<br>**Genotype**<br>**Diet X genotype**	8.640<br>0.266<br>1.083	**0.0057**<br>0.6094<br>0.3049
	Female	2.3 ± 0.1	2.5 ± 0.1	4.07 ± 0.4	4.004 ± 0.3	**Diet**<br>**Genotype**<br>**Diet X genotype**	43.60<br>0.066<br>0.265	**<0.0001**<br>0.7994<br>0.6098
PC aa C40:6	Male	21.09 ± 0.9	21.6 ± 0.9	29.5 ± 2.3	32.7 ± 2.2	**Diet**<br>**Genotype**<br>**Diet X genotype**	31.12<br>1.117<br>0.631	**<0.0001**<br>0.2979<br>0.4325
	Female	21.7 ± 2.2	21.8 ± 2.1	36.5 ± 2.2	39.3 ± 3.5	**Diet**<br>**Genotype**<br>**Diet X genotype**	39.40<br>0.313<br>0.292	**<0.0001**<br>0.5794<br>0.5923
PC aa C42:0	Male	0.2 ± 0.01	0.2 ± 0.02	0.3 ± 0.04	0.3 ± 0.03	**Diet**<br>**Genotype**<br>**Diet X genotype**	29.21<br>1.136<br>0.778	**<0.0001**<br>0.2935<br>0.3836
	Female	0.2 ± 0.02	0.2 ± 0.02	0.4 ± 0.04	0.4 ± 0.04	**Diet**<br>**Genotype**<br>**Diet X genotype**	28.88<br>0.969<br>0.951	**<0.0001**<br>0.3318<br>0.3362
PC aa C42:1	Male	0.2 ± 0.03	0.2 ± 0.02	0.2 ± 0.02	0.3 ± 0.02	**Diet**<br>**Genotype**<br>**Diet X genotype**	2.443<br>0.014<br>3429	0.1271<br>0.9081<br>0.0725

	Female	0.2 ± 0.3	0.2 ± 0.02	0.3 ± 0.03	0.3 ± 0.03	**Diet**<br>**Genotype**<br>**Diet X genotype**	10.76<br>1.962<br>0.3095	**0.0024**<br>0.1701<br>0.5816
PC aa C42:2	Male	0.4 ± 0.03	0.4 ± 0.02	0.4 ± 0.03	0.5 ± 0.03	**Diet**<br>**Genotype**<br>**Diet X genotype**	0.923<br>1.089<br>1.672	0.3431<br>0.3036<br>0.2043
	Female	0.3 ± 0.01	0.3 ± 0.04	0.4 ± 0.05	0.4 ± 0.02	**Diet**<br>**Genotype**<br>**Diet X genotype**	6.061<br>0.043<br>8.631e-005	**0.0189**<br>0.8368<br>0.9926
PC aa C42:4	Male	0.2 ± 0.02	0.2 ± 0.02	0.3 ± 0.04	0.4 ± 0.02	**Diet**<br>**Genotype**<br>**Diet X genotype**	13.61<br>0.008<br>0.112	**0.0007**<br>0.9301<br>0.7395
	Female	0.2 ± 0.01	0.2 ± 0.02	0.3 ± 0.02	0.3 ± 0.03	**Diet**<br>**Genotype**<br>**Diet X genotype**	28.29<br>1.589<br>0.188	**<0.0001**<br>0.2158<br>0.6674
PC aa C42:5	Male	0.4 ± 0.05	0.4 ± 0.03	0.5 ± 0.04	0.4 ± 0.04	**Diet**<br>**Genotype**<br>**Diet X genotype**	0.106<br>1.328<br>0.048	0.7467<br>0.2568<br>0.8278
	Female	0.3 ± 0.02	0.2 ± 0.03	0.3 ± 0.03	0.3 ± 0.03	**Diet**<br>**Genotype**<br>**Diet X genotype**	0.535<br>0.545<br>2.124	0.4695<br>0.4653<br>0.1539
PC aa C42:6	Male	0.7 ± 0.08	0.6 ± 0.04	0.7 ± 0.04	0.9 ± 0.07	**Diet**<br>**Genotype**<br>**Diet X genotype**	9.292<br>0.288<br>2.774	**0.0043**<br>0.5948<br>0.1045
	Female	0.2 ± 0.02	0.2 ± 0.03	0.3 ± 0.03	0.3 ± 0.03	**Diet**<br>**Genotype**<br>**Diet X genotype**	5.891<br>0.087<br>0.148	**0.0207**<br>0.7695<br>0.7029
Phosphatidycholine acyl-alkyls								
PC ae C30:0	Male	0.2 ± 0.03	0.2 ± 0.02	0.3 ± 0.03	0.3 ± 0.02	**Diet**<br>**Genotype**<br>**Diet X genotype**	3.256<br>0.014<br>1.235	0.0795<br>0.9081<br>0.2739
	Female	0.2 ± 0.03	0.15 ± 0.02	0.3 ± 0.02	0.3 ± 0.02	**Diet**<br>**Genotype**	5.744<br>5.869	**0.0220**<br>**0.0207**

						**Diet X genotype**	1.098	0.3019
PC ae C30:1	Male	0.3 ± 0.06	0.2 ± 0.04	0.3 ± 0.05	0.3 ± 0.06	**Diet**	1.907	0.1758
						**Genotype**	0.060	0.8082
						**Diet X genotype**	0.519	0.4759
	Female	0.3 ± 0.06	0.12 ± 0.03	0.19 ± 0.05	0.2 ± 0.03	**Diet**	0.034	0.8539
						**Genotype**	2.447	0.1273
						**Diet X genotype**	2.702	0.1097
PC ae C30:2	Male	0.123 ± 0.04	0.1 ± 0.008	0.14 ± 0.02	0.15 ± 0.01	**Diet**	3.107	0.0864
						**Genotype**	0.162	0.6902
						**Diet X genotype**	1.792	0.1891
	Female	0.12 ± 0.01	0.09 ± 0.01	0.16 ± 0.01	0.17 ± 0.01	**Diet**	19.04	**0.0001**
						**Genotype**	0.412	0.5253
						**Diet X genotype**	2.191	0.1478
PC ae C32:1	Male	0.8 ± 0.1	0.7 ± 0.03	0.9 ± 0.06	1.06 ± 0.08	**Diet**	4.885	**0.0335**
						**Genotype**	0.459	0.5025
						**Diet X genotype**	4.203	**0.0477**
	Female	0.7 ± 0.07	0.5 ± 0.03	0.8 ± 0.08	0.8 ± 0.05	**Diet**	9.496	**0.0040**
						**Genotype**	0.666	0.4199
						**Diet X genotype**	2.206	0.1465
PC ae C32:2	Male	0.4 ± 0.03	0.3 ± 0.03	0.5 ± 0.04	0.5 ± 0.03	**Diet**	22.11	**<0.0001**
						**Genotype**	0.110	0.7427
						**Diet X genotype**	0.882	0.3541
	Female	0.3 ± 0.03	0.3 ± 0.02	0.4 ± 0.04	0.3 ± 0.02	**Diet**	5.462	**0.0253**
						**Genotype**	2.968	0.0938
						**Diet X genotype**	0.502	0.4832
PC ae C34:0	Male	0.5 ± 0.05	0.4 ± 0.05	0.5 ± 0.04	0.7 ± 0.05	**Diet**	16.41	**0.0003**
						**Genotype**	2.412	0.1292
						**Diet X genotype**	5.255	**0.0278**
	Female	0.5 ± 0.04	0.4 ± 0.03	0.7 ± 0.06	0.6 ± 0.06	**Diet**	12.90	**0.0010**
						**Genotype**	0.439	0.5122
						**Diet X genotype**	0.056	0.8148
PC ae C34:1	Male	2.8 ± 0.3	3.3 ± 0.2	4.4 ± 0.4	4.5 ± 0.1	**Diet**	24.48	**<0.0001**
						**Genotype**	1.364	0.2506
						**Diet X genotype**	0.361	0.5517

	Female	1.9 ± 0.2	1.9 ± 0.1	3.4 ± 0.2	3.5 ± 0.2	**Diet**<br>**Genotype**<br>**Diet X genotype**	60.13<br>0.250<br>0.027	**<0.0001**<br>0.6200<br>0.8701
PC ae C34:2	Male	2.6 ± 0.2	2.9 ± 0.2	3.08 ± 0.2	3.8 ± 0.2	**Diet**<br>**Genotype**<br>**Diet X genotype**	10.73<br>6.232<br>0.695	**0.0023**<br>**0.0173**<br>0.4099
	Female	1.5 ± 0.2	1.7 ± 0.09	1.6 ± 0.2	1.7 ± 0.1	**Diet**<br>**Genotype**<br>**Diet X genotype**	0.294<br>0.455<br>0.028	0.5912<br>0.5043<br>0.8689
PC ae C34:3	Male	0.8 ± 0.09	1.0 ± 0.07	1.2 ± 0.06	1.3 ± 0.1	**Diet**<br>**Genotype**<br>**Diet X genotype**	13.58<br>2.393<br>0.120	**0.0008**<br>0.1308<br>0.7310
	Female	0.6 ± 0.07	0.6 ± 0.04	0.9 ± 0.1	0.8 ± 0.06	**Diet**<br>**Genotype**<br>**Diet X genotype**	12.03<br>0.557<br>1.216	**0.0014**<br>0.4605<br>0.2777
PC ae C36:0	Male	0.8 ± 0.06	0.8 ± 0.03	1.1 ± 0.1	1.2 ± 0.07	**Diet**<br>**Genotype**<br>**Diet X genotype**	33.62<br>0.890<br>0.330	**<0.0001**<br>0.3517<br>0.5690
	Female	0.6 ± 0.03	0.6 ± 0.06	0.9 ± 0.09	1.07 ± 0.08	**Diet**<br>**Genotype**<br>**Diet X genotype**	32.43<br>1.199<br>0.580	**<0.0001**<br>0.2810<br>0.4516
PC ae C36:1	Male	3.0 ± 0.2	2.9 ± 0.1	4.9 ± 0.3	5.6 ± 0.3	**Diet**<br>**Genotype**<br>**Diet X genotype**	90.60<br>1.530<br>2.609	**<0.0001**<br>0.2242<br>0.1150
	Female	1.9 ± 0.2	2.03 ± 0.1	3.5 ± 0.3	3.4 ± 0.2	**Diet**<br>**Genotype**<br>**Diet X genotype**	46.35<br>0.012<br>0.117	**<0.0001**<br>0.9132<br>0.7341
PC ae C36:2	Male	8.6 ± 0.6	9.8 ± 0.7	10.2 ± 0.8	11.5 ± 0.6	**Diet**<br>**Genotype**<br>**Diet X genotype**	5.394<br>3.106<br>0.013	**0.0261**<br>0.0868<br>0.9092
	Female	5.9 ± 0.7	7.07 ± 0.6	6.6 ± 0.6	7.0 ± 0.4	**Diet**<br>**Genotype**<br>**Diet X genotype**	0.330<br>1.806<br>0.609	0.5691<br>0.1876<br>0.4403
PC ae C36:3	Male	1.8 ± 0.18	1.8 ± 0.07	2.3 ± 0.2	2.4 ± 0.1	**Diet**	12.04	**0.0014**

						**Genotype**	0.048	0.8280
						**Diet X genotype**	0.002	0.9653
	Female	1.0 ± 0.1	1.0 ± 0.08	1.4 ± 0.1	1.3 ± 0.08	**Diet**	13.78	**0.0007**
						**Genotype**	0.260	0.6135
						**Diet X genotype**	0.172	0.6810
PC ae C36:4	Male	1.8 ± 0.2	2.3 ± 0.1	3.7 ± 0.4	3.8 ± 0.3	**Diet**	46.97	**<0.0001**
						**Genotype**	1.297	0.2623
						**Diet X genotype**	0.781	0.3827
	Female	1.6 ± 0.2	1.5 ± 0.09	3.0 ± 0.2	3.0 ± 0.2	**Diet**	55.93	**<0.0001**
						**Genotype**	0.079	0.7805
						**Diet X genotype**	0.09	0.7644
PC ae C36:5	Male	1.2 ± 0.08	1.4 ± 0.1	2.2 ± 0.2	2.4 ± 0.1	**Diet**	54.90	**<0.0001**
						**Genotype**	1.220	0.2769
						**Diet X genotype**	0.018	0.8948
	Female	1.2 ± 0.1	1.14 ± 0.08	2.5 ± 0.3	2.07 ± 0.1	**Diet**	38.31	**<0.0001**
						**Genotype**	1.361	0.2513
						**Diet X genotype**	1.167	0.2873
PC ae C38:0	Male	4.09 ± 0.4	4.2 ± 0.2	3.5 ± 0.5	4.07 ± 0.3	**Diet**	0.890	0.3517
						**Genotype**	0.785	0.3815
						**Diet X genotype**	0.462	0.5009
	Female	1.8 ± 0.1	1.9 ± 0.2	3.3 ± 0.3	3.2 ± 0.3	**Diet**	42.72	**<0.0001**
						**Genotype**	0.014	0.9077
						**Diet X genotype**	0.065	0.8004
PC ae C38:1	Male	1.2 ± 0.1	1.05 ± 0.09	1.9 ± 0.2	2.3 ± 0.4	**Diet**	20.40	**<0.0001**
						**Genotype**	0.501	0.4838
						**Diet X genotype**	1.630	0.2099
	Female	0.6 ± 0.08	0.8 ± 0.1	1.6 ± 0.1	1.6 ± 0.2	**Diet**	46.09	**<0.0001**
						**Genotype**	0.122	0.7286
						**Diet X genotype**	0.590	0.4474
PC ae C38:2	Male	6.9 ± 0.9	9.2 ± 1.0	6.0 ± 0.5	6.7 ± 0.5	**Diet**	5.135	**0.0296**
						**Genotype**	4.168	**0.0486**
						**Diet X genotype**	1.095	0.3024
	Female	4.02 ± 0.4	5.4 ± 0.6	3.2 ± 0.3	3.4 ± 0.3	**Diet**	10.19	**0.0030**
						**Genotype**	3.295	0.0781

						**Diet X genotype**	1.762	0.1930
PC ae C38:3	Male	1.8 ± 0.2	1.9 ± 0.1	2.0 ± 0.1	2.4 ± 0.1	**Diet**	6.226	**0.0173**
						**Genotype**	4.182	**0.0482**
						**Diet X genotype**	1.291	0.2633
	Female	1.3 ± 0.1	1.4 ± 0.2	1.9 ± 0.1	1.9 ± 0.13	**Diet**	12.63	**0.0011**
						**Genotype**	0.268	0.6083
						**Diet X genotype**	0.173	0.6798
PC ae C38:4	Male	2.5 ± 0.2	2.7 ± 0.1	4.4 ± 0.4	4.3 ± 0.2	**Diet**	48.60	**<0.0001**
						**Genotype**	0.033	0.8561
						**Diet X genotype**	0.322	0.5740
	Female	2.7 ± 0.3	2.6 ± 0.2	4.2 ± 0.4	4.3 ± 0.2	**Diet**	33.50	**<0.0001**
						**Genotype**	0.0009	0.9758
						**Diet X genotype**	0.065	0.8001
PC ae C38:5	Male	1.6 ± 0.1	1.9 ± 0.1	3.2 ± 0.2	3.6 ± 0.1	**Diet**	126.5	**<0.0001**
						**Genotype**	6.162	**0.0178**
						**Diet X genotype**	0.008	0.9309
	Female	1.4 ± 0.1	1.4 ± 0.1	3.3 ± 0.2	3.2 ± 0.2	**Diet**	98.01	**<0.0001**
						**Genotype**	0.0007	0.9794
						**Diet X genotype**	0.038	0.8461
PC ae C38:6	Male	1.2 ± 0.1	1.2 ± 0.08	1.9 ± 0.2	2.002 ± 0.1	**Diet**	27.00	**<0.0001**
						**Genotype**	0.557	0.4604
						**Diet X genotype**	0.124	0.7270
	Female	0.9 ± 0.07	0.8 ± 0.07	1.6 ± 0.2	1.6 ± 0.09	**Diet**	40.19	**<0.0001**
						**Genotype**	0.407	0.5277
						**Diet X genotype**	0.313	0.5797
PC ae C40:1	Male	2.6 ± 0.2	2.3 ± 0.1	4.1 ± 0.5	3.8 ± 0.3	**Diet**	23.28	**<0.0001**
						**Genotype**	0.761	0.3889
						**Diet X genotype**	0.019	0.8908
	Female	1.7 ± 0.1	1.7 ± 0.2	4.05 ± 0.3	3.7 ± 0.4	**Diet**	51.64	**<0.0001**
						**Genotype**	0.517	0.4767
						**Diet X genotype**	0.366	0.5494
PC ae C40:2	Male	0.7 ± 0.02	0.8 ± 0.05	0.9 ± 0.06	0.9 ± 0.1	**Diet**	6.374	**0.0161**
						**Genotype**	0.728	0.3992
						**Diet X genotype**	0.044	0.8344

	Female	0.6 ± 0.04	0.5 ± 0.05	1.01 ± 0.08	0.8 ± 0.07	**Diet**<br>**Genotype**<br>**Diet X genotype**	34.05<br>5.380<br>1.733	**<0.0001**<br>**0.0263**<br>0.1966
PC ae C40:3	Male<br><br><br>Female	0.8 ± 0.1<br><br><br>0.7 ± 0.06	0.7 ± 0.05<br><br><br>0.6 ± 0.06	0.8 ± 0.07<br><br><br>1.0 ± 0.06	1.0 ± 0.05<br><br><br>1.0 ± 0.07	**Diet**<br>**Genotype**<br>**Diet X genotype**<br>**Diet**<br>**Genotype**<br>**Diet X genotype**	2.820<br>0.071<br>1.991<br>26.32<br>0.062<br>0.002	0.1018<br>0.7908<br>0.1668<br>**<0.0001**<br>0.8047<br>0.9686
PC ae C40:4	Male<br><br><br>Female	2.02 ± 0.1<br><br><br>1.8 ± 0.1	2.1 ± 0.2<br><br><br>1.8 ± 0.2	2.5 ± 0.2<br><br><br>2.03 ± 0.1	2.4 ± 0.2<br><br><br>1.9 ± 0.1	**Diet**<br>**Genotype**<br>**Diet X genotype**<br>**Diet**<br>**Genotype**<br>**Diet X genotype**	4.018<br>0.004<br>0.540<br>1.129<br>0.136<br>0.555	0.0526<br>0.9486<br>0.4671<br>0.2953<br>0.7148<br>0.4613
PC ae C40 :5	Male<br><br><br>Female	0.8 ± 0.06<br><br><br>0.8 ± 0.05	0.8 ± 0.06<br><br><br>0.7 ± 0.06	1.1 ± 0.07<br><br><br>1.3 ± 0.1	1.2 ± 0.07<br><br><br>1.2 ± 0.09	**Diet**<br>**Genotype**<br>**Diet X genotype**<br>**Diet**<br>**Genotype**<br>**Diet X genotype**	29.12<br>0.226<br>0.491<br>41.43<br>0.817<br>0.0009	**<0.0001**<br>0.6372<br>0.4882<br>**<0.0001**<br>0.3723<br>0.9750
PC ae C40:6	Male<br><br><br>Female	1.6 ± 0.2<br><br><br>1.5 ± 0.1	1.5 ± 0.08<br><br><br>1.5 ± 0.2	2.1 ± 0.2<br><br><br>2.2 ± 0.2	2.3 ± 0.1<br><br><br>2.5 ± 0.2	**Diet**<br>**Genotype**<br>**Diet X genotype**<br>**Diet**<br>**Genotype**<br>**Diet X genotype**	16.96<br>0.033<br>0.808<br>24.56<br>0.483<br>0.361	**0.0002**<br>0.8560<br>0.3748<br>**<0.0001**<br>0.4918<br>0.5518
PC ae C42:0	Male<br><br><br>Female	1.6 ± 0.1<br><br><br>1.0 ± 0.06	1.5 ± 0.1<br><br><br>0.9 ± 0.05	1.3 ± 0.1<br><br><br>1.2 ± 0.09	1.2 ± 0.06<br><br><br>1.06 ± 0.07	**Diet**<br>**Genotype**<br>**Diet X genotype**<br>**Diet**<br>**Genotype**<br>**Diet X genotype**	11.02<br>0.265<br>0.060<br>6.504<br>1.383<br>0.089	**0.0021**<br>0.6101<br>0.8077<br>**0.0153**<br>0.2476<br>0.7668
PC ae C42:1	Male	0.7 ± 0.06	0.6 ± 0.06	0.7 ± 0.09	0.8 ± 0.08	**Diet**	2.994	0.0922

						**Genotype**	0.031	0.8620
						**Diet X genotype**	0.548	0.4639
	Female	0.6 ± 0.04	0.5 ± 0.07	0.9 ± 0.06	0.9 ± 0.06	**Diet**	26.50	**<0.0001**
						**Genotype**	2.624	0.1142
						**Diet X genotype**	0.078	0.7816
PC ae C42:2	Male	0.6 ± 0.06	0.5 ± 0.03	0.8 ± 0.07	0.8 ± 0.07	**Diet**	27.82	**<0.0001**
						**Genotype**	0.034	0.8541
						**Diet X genotype**	2.409	0.1294
	Female	0.5 ± 0.05	0.4 ± 0.07	0.9 ± 0.08	0.9 ± 0.08	**Diet**	31.51	**<0.0001**
						**Genotype**	0.032	0.8599
						**Diet X genotype**	0.093	0.7627
PC ae C42:3	Male	1.09 ± 0.1	0.9 ± 0.07	1.7 ± 0.2	1.7 ± 0.2	**Diet**	23.70	**<0.0001**
						**Genotype**	0.851	0.3624
						**Diet X genotype**	0.068	0.7953
	Female	0.8 ± 0.06	0.7 ± 0.08	1.7 ± 0.2	1.7 ± 0.2	**Diet**	43.40	**<0.0001**
						**Genotype**	0.278	0.6016
						**Diet X genotype**	0.193	0.6632
PC ae C42:4	Male	0.3 ± 0.04	0.3 ± 0.03	0.4 ± 0.04	0.5 ± 0.02	**Diet**	4.132	**0.0495**
						**Genotype**	1.313	0.2594
						**Diet X genotype**	4.223	**0.0472**
	Female	0.3 ± 0.03	0.3 ± 0.03	0.4 ± 0.03	0.4 ± 0.05	**Diet**	12.34	**0.0012**
						**Genotype**	0.148	0.7028
						**Diet X genotype**	0.150	0.7012
PC ae C44:3	Male	0.2 ± 0.02	0.1 ± 0.01	0.2 ± 0.03	0.2 ± 0.02	**Diet**	10.46	**0.0026**
						**Genotype**	1.083	0.3050
						**Diet X genotype**	0.606	0.4414
	Female	0.2 ± 0.01	0.1 ± 0.02	0.2 ± 0.02	0.3 ± 0.02	**Diet**	30.31	**<0.0001**
						**Genotype**	0.010	0.9208
						**Diet X genotype**	6.005	**0.0194**
PC ae C44:4	Male	0.2 ± 0.03	0.18 ± 0.01	0.2 ± 0.03	0.2 ± 0.03	**Diet**	0.175	0.6783
						**Genotype**	0.142	0.7089
						**Diet X genotype**	2.755	0.1056
	Female	0.2 ± 0.02	0.18 ± 0.02	0.3 ± 0.03	0.3 ± 0.02	**Diet**	25.67	**0.0004**
						**Genotype**	0.353	0.5561

						**Diet X genotype**	0.114	0.7381
PC ae C44:5	Male	0.18 ± 0.02	0.17 ± 0.01	0.2 ± 0.03	0.3 ± 0.03	**Diet**	9.813	**0.0034**
						**Genotype**	0.095	0.7594
						**Diet X genotype**	0.321	0.5746
	Female	0.17 ± 0.02	0.16 ± 0.01	0.2 ± 0.03	0.3 ± 0.03	**Diet**	8.079	**0.0074**
						**Genotype**	0.150	0.7012
						**Diet X genotype**	0.370	0.5472
PC ae C44:6	Male	0.2 ± 0.05	0.17 ± 0.01	0.2 ± 0.02	0.17 ± 0.02	**Diet**	0.303	0.5853
						**Genotype**	2.751	0.1059
						**Diet X genotype**	0.242	0.6260
	Female	0.18 ± 0.01	0.1 ± 0.02	0.18 ± 0.02	0.2 ± 0.02	**Diet**	6.223	**0.0175**
						**Genotype**	0.019	0.8900
						**Diet X genotype**	5.930	**0.0201**
**Sphingolipids**								
SM(OH) C14:1	Male	0.6 ± 0.04	0.6 ± 0.04	1.08 ± 0.09	1.08 ± 0.04	**Diet**	71.59	**<0.0001**
						**Genotype**	0.218	0.6431
						**Diet X genotype**	0.191	0.6651
	Female	0.7 ± 0.04	0.7 ± 0.05	1.5 ± 0.1	1.6 ± 0.1	**Diet**	101.9	**<0.0001**
						**Genotype**	0.711	0.4048
						**Diet X genotype**	0.346	0.5600
SM(OH) C16:1	Male	0.09 ± 0.01	0.09 ± 0.009	0.2 ± 0.02	0.17 ± 0.01	**Diet**	32.51	**<0.0001**
						**Genotype**	0.005	0.9438
						**Diet X genotype**	0.019	0.8907
	Female	0.14 ± 0.01	0.14 ± 0.02	0.3 ± 0.04	0.3 ± 0.02	**Diet**	50.66	**<0.0001**
						**Genotype**	0.010	0.9207
						**Diet X genotype**	0.033	0.8570
SM(OH) C22:1	Male	1.0 ± 0.4	0.6 ± 0.06	0.8 ± 0.06	0.8 ± 0.06	**Diet**	0.092	0.7640
						**Genotype**	1.118	0.2975
						**Diet X genotype**	0.985	0.3276
	Female	0.7 ± 0.06	0.6 ± 0.08	0.8 ± 0.06	0.9 ± 0.06	**Diet**	6.030	**0.0192**
						**Genotype**	0.137	0.7139
						**Diet X genotype**	3.551	0.0678
SM(OH) C22:2	Male	0.7 ± 0.05	0.7 ± 0.07	0.7 ± 0.07	0.7 ± 0.05	**Diet**	0.060	0.8073
						**Genotype**	0.908	0.3471

						**Diet X genotype**	0.037	0.8484
	Female	0.5 ± 0.03	0.5 ± 0.03	0.6 ± 0.04	0.6 ± 0.02	**Diet**	11.88	**0.0015**
						**Genotype**	0.158	0.6938
						**Diet X genotype**	0.610	0.4401
SM(OH) C24:1	Male	0.13 ± 0.01	0.1 ± 0.01	0.13 ± 0.03	0.15 ± 0.02	**Diet**	1.242	0.2729
						**Genotype**	0.043	0.8368
						**Diet X genotype**	1.344	0.2544
	Female	0.11 ± 0.01	0.1 ± 0.01	0.14 ± 0.02	0.2 ± 0.01	**Diet**	19.83	**<0.0001**
						**Genotype**	4.475	**0.0416**
						**Diet X genotype**	7.174	**0.0112**
SM C16:0	Male	6.3 ± 0.5	6.7 ± 0.3	9.3 ± 0.5	10.3 ± 0.4	**Diet**	62.19	**<0.0001**
						**Genotype**	2.505	0.1225
						**Diet X genotype**	0.678	0.4158
	Female	6.7 ± 0.3	7.3 ± 0.4	12.9 ± 0.8	14.3 ± 0.7	**Diet**	112.4	**<0.0001**
						**Genotype**	2.381	0.1318
						**Diet X genotype**	0.495	0.4863
SM C16:1	Male	1.3 ± 0.1	1.3 ± 0.09	1.9 ± 0.1	2.2 ± 0.1	**Diet**	2.544	**<0.0001**
						**Genotype**	2.661	0.1118
						**Diet X genotype**	46.87	0.1197
	Female	0.8 ± 0.06	0.8 ± 0.04	1.6 ± 0.1	1.6 ± 0.07	**Diet**	91.10	**<0.0001**
						**Genotype**	0.014	0.9079
						**Diet X genotype**	0.0008	0.9781
SM C18:0	Male	2.09 ± 0.3	2.3 ± 0.1	4.3 ± 0.3	5.5 ± 0.3	**Diet**	108.5	**<0.0001**
						**Genotype**	7.721	**0.0086**
						**Diet X genotype**	3.852	0.0575
	Female	5.8 ± 0.7	5.3 ± 0.6	16.0 ± 2.0	15.8 ± 0.9	**Diet**	75.91	**<0.0001**
						**Genotype**	0.090	0.7656
						**Diet X genotype**	0.033	0.8560
SM C18:1	Male	0.19 ± 0.02	0.2 ± 0.02	0.4 ± 0.02	0.5 ± 0.05	**Diet**	66.39	**<0.0001**
						**Genotype**	3.139	0.0852
						**Diet X genotype**	1.363	0.2510
	Female	0.3 ± 0.03	0.3 ± 0.03	0.9 ± 0.1	0.8 ± 0.05	**Diet**	71.61	**<0.0001**
						**Genotype**	0.709	0.4054
						**Diet X genotype**	0.572	0.4547

SM C20:2	Male	0.03 ± 0.009	0.02 ± 0.005	0.02 ± 0.004	0.03 ± 0.004	**Diet**	0.090	0.7658
						**Genotype**	0.877	0.3569
						**Diet X genotype**	2.554	0.1212
	Female	0.03 ± 0.005	0.03 ± 0.004	0.05 ± 0.007	0.05 ± 0.007	**Diet**	5.213	**0.0294**
						**Genotype**	0.030	0.8633
						**Diet X genotype**	0.0009	0.9758
SM C22:3	Male	0.1 ± 0.03	0.07 ± 0.03	0.3 ± 0.04	0.2 ± 0.03	**Diet**	23.69	**<0.0001**
						**Genotype**	1.884	0.1784
						**Diet X genotype**	0.381	0.5408
	Female	0.07 ± 0.02	0.06 ± 0.02	0.2 ± 0.03	0.2 ± 0.04	**Diet**	28.85	**<0.0001**
						**Genotype**	1.064	0.3093
						**Diet X genotype**	0.345	0.5610
SM C24:0	Male	2.5 ± 0.1	2.7 ± 0.1	2.9 ± 0.1	3.3 ± 0.1	**Diet**	15.66	**0.0004**
						**Genotype**	5.197	**0.0288**
						**Diet X genotype**	0.095	0.7596
	Female	1.4 ± 0.08	1.6 ± 0.1	2.01 ± 0.1	1.9 ± 0.09	**Diet**	15.34	**0.0004**
						**Genotype**	0.470	0.4975
						**Diet X genotype**	1.573	0.2181
SM C24:1	Male	4.3 ± 0.3	4.3 ± 0.3	3.5 ± 0.3	3.4 ± 0.2	**Diet**	11.36	**0.0018**
						**Genotype**	0.027	0.8714
						**Diet X genotype**	0.087	0.7701
	Female	4.3 ± 0.2	4.08 ± 0.3	3.9 ± 0.3	4.2 ± 0.2	**Diet**	0.264	0.6107
						**Genotype**	0.014	0.9057
						**Diet X genotype**	0.980	0.3291
SM C26:0	Male	0.08 ± 0.06	0.02 ± 0.008	0.04 ± 0.009	0.04 ± 0.008	**Diet**	0.287	0.5957
						**Genotype**	1.139	0.2930
						**Diet X genotype**	0.947	0.3371
	Female	0.02 ± 0.006	0.05 ± 0.01	0.04 ± 0.009	0.04 ± 0.008	**Diet**	0.040	0.8420
						**Genotype**	1.498	0.2292
						**Diet X genotype**	1.138	0.2934
SM C26:1	Male	0.04 ± 0.009	0.05 ± 0.01	0.04 ± 0.009	0.04 ± 0.008	**Diet**	0.660	0.4219
						**Genotype**	0.429	0.5166
						**Diet X genotype**	0.457	0.5036
	Female	0.03 ± 0.009	0.02 ± 0.009	0.06 ± 0.01	0.05 ± 0.012	**Diet**	6.462	**0.0156**

						**Genotype**	0.245	0.6237
						**Diet X genotype**	0.014	0.9051
**Monosaccharides**								
Hexoses (H1)	Male	7670.2 ± 318.7	7696.7± 289.7	7784.7 ± 374.2	8668.7 ± 403.2	**Diet**	2.387	0.1313
						**Genotype**	1.676	0.2039
						**Diet X genotype**	1.487	0.2308
	Female	6011.3 ± 308.2	5848.8± 197.6	7291.1 ± 399.5	7319.2 ± 323.9	**Diet**	18.31	**0.0001**
						**Genotype**	0.044	0.8355
						**Diet X genotype**	0.088	0.7685
**Metabolite ratios**								
Monounsaturated fatty acids ((MUFA (PC))	Male	249.7 ± 9.6	265.5 ± 18.2	350.3 ± 23.7	383.5 ± 11.1	**Diet**	41.48	**<0.0001**
						**Genotype**	2.086	0.1575
						**Diet X genotype**	0.262	0.6122
	Female	110.7 ± 10.02	136.03± 9.7	245.5 ± 17.5	260.1 ± 16.1	**Diet**	85.36	**<0.0001**
						**Genotype**	2.034	0.1627
						**Diet X genotype**	0.148	0.7032
Polyunsaturated fatty acids ((PUFA (PC))	Male	1012.7 ± 42.9	1131.6± 51.0	1506.3 ± 98.9	1683 ± 64.1	**Diet**	57.52	**<0.0001**
						**Genotype**	4.604	**0.0389**
						**Diet X genotype**	0.176	0.6776
	Female	656.2 ± 52.2	736 ± 49.2	1225.9 ± 90.8	1249.3 ± 90.4	**Diet**	52.29	**<0.0001**
						**Genotype**	0.478	0.4941
						**Diet X genotype**	0.143	0.7080
Saturated fatty acids ((SFA (PC))	Male	31.7 ± 1.5	33.6 ± 2.1	32.7 ± 2.5	44.6 ± 2.5	**Diet**	5.857	**0.0208**
						**Genotype**	7.728	**0.0087**
						**Diet X genotype**	4.203	**0.0479**
	Female	27.7 ± 1.6	27.4 ± 1.7	40.2 ± 3.2	41.4 ± 3.1	**Diet**	26.66	**<0.0001**
						**Genotype**	0.031	0.8609
						**Diet X genotype**	0.081	0.7772
C2/C0	Male	0.5 ± 0.05	0.6 ± 0.05	0.4 ± 0.01	0.5 ± 0.05	**Diet**	6.818	**0.0132**
						**Genotype**	2.513	0.1219
						**Diet X genotype**	0.107	0.7457
	Female	0.9 ± 0.1	0.6 ± 0.05	0.5 ± 0.04	0.5 ± 0.03	**Diet**	18.23	**0.0001**
						**Genotype**	3.867	0.0572
						**Diet X genotype**	4.320	**0.0451**

C3/C0	Male	0.019 ± 0.001	0.026 ± 0.002	0.021 ± 0.002	0.024 ± 0.001	**Diet**	0.010	0.9204
						**Genotype**	10.42	**0.0027**
						**Diet X genotype**	1.590	0.2154
	Female	0.028 ± 0.001	0.027 ± 0.002	0.021 ± 0.002	0.023 ± 0.001	**Diet**	11.13	**0.0020**
						**Genotype**	0.119	0.7318
						**Diet X genotype**	0.485	0.4907
C4/C0	Male	0.029 ± 0.004	0.032 ± 0.003	0.030 ± 0.002	0.029 ± 0.002	**Diet**	0.071	0.7909
						**Genotype**	0.071	0.7911
						**Diet X genotype**	0.399	0.5314
	Female	0.034 ± 0.003	0.032 ± 0.003	0.031 ± 0.002	0.033 ± 0.002	**Diet**	0.088	0.7685
						**Genotype**	0.066	0.7992
						**Diet X genotype**	0.570	0.4552
C5/C0	Male	0.009 ± 0.0007	0.010 ± 0.0007	0.015 ± 0.001	0.015 ± 0.001	**Diet**	25.65	**<0.0001**
						**Genotype**	0.188	0.6669
						**Diet X genotype**	0.499	0.4847
	Female	0.013 ± 0.001	0.012 ± 0.001	0.016 ± 0.002	0.017 ± 0.001	**Diet**	7.222	**0.0109**
						**Genotype**	0.003	0.9547
						**Diet X genotype**	0.879	0.3548
Fisher ratio	Male	2.003 ± 0.06	2.1 ± 0.08	2.8 ± 0.1	2.5 ± 0.1	**Diet**	32.22	**<0.0001**
						**Genotype**	0.446	0.5086
						**Diet X genotype**	2.785	0.1040
	Female	1.7 ± 0.06	1.8 ± 0.07	1.9 ± 0.1	1.8 ± 0.1	**Diet**	0.487	0.4897
						**Genotype**	0.012	0.9123
						**Diet X genotype**	0.734	0.3973
Branched-chain amino acids (BCAA)	Male	452.3 ± 37.0	476.3 ± 46.2	608.4 ± 38.9	663 ± 66.0	**Diet**	12.78	**0.0011**
						**Genotype**	0.672	0.4182
						**Diet X genotype**	0.102	0.7513
	Female	409.5 ± 34.2	450.8± 24.2	410.5 ± 26.6	485.4 ± 56.4	**Diet**	0.217	0.6442
						**Genotype**	2.309	0.1376
						**Diet X genotype**	0.193	0.6628
Aromatic amino acids (AAA)	Male	225.9 ± 17.8	226.4 ± 18.2	226.6 ± 19.7	299.1 ± 42.1	**Diet**	1.858	0.1816
						**Genotype**	1.837	0.1839
						**Diet X genotype**	1.786	0.1900
	Female	236.5 ± 21.5	245.1 ± 9.3	219.8 ± 12.7	263.4 ± 17.8	**Diet**	0.002	0.9615

						**Genotype**	2.552	0.1191
						**Diet X genotype**	1.146	0.2917
Glucogenic amino acids	Male	751.4 ± 61.8	690.4 ± 61.0	683 ± 44.3	887.6 ± 62.5	**Diet**	1.240	0.2730
						**Genotype**	1.542	0.2226
						**Diet X genotype**	5.280	**0.0277**
	Female	697.5 ± 29.6	570.6± 16.8	657.9 ± 40.0	776.2 ± 46.9	**Diet**	12.66	**0.0011**
						**Genotype**	1.278	0.2659
						**Diet X genotype**	4.655	**0.0379**
Ketogenic amino acids	Male	447.3 ± 81.1	472.2 ± 83.5	558.7 ± 51.03	896.2 ± 158.08	**Diet**	6.968	**0.0122**
						**Genotype**	3.193	0.0824
						**Diet X genotype**	2.375	0.1321
	Female	360.4 ± 20.6	376.3 ± 10.3	451.7 ± 35.2	501.4 ± 44.6	**Diet**	11.84	**0.0015**
						**Genotype**	1.091	0.3033
						**Diet X genotype**	0.288	0.5947
Essential amino acids	Male	1149.7 ± 125.5	1017.1 ± 58.04	1391.7 ± 103.6	1680.3 ± 170.4	**Diet**	14.08	**0.0007**
						**Genotype**	0.419	0.5221
						**Diet X genotype**	3.048	0.0901
	Female	1016.6 ± 65.6	1079.7 ± 39.5	1114.7 ± 79.0	1297.4 ± 104.07	**Diet**	4.173	**0.0487**
						**Genotype**	2.527	0.1209
						**Diet X genotype**	0.599	0.4443
Non-essential amino acids	Male	2127.6 ± 116.8	2021.3 ± 98.2	1974.6 ± 91.15	2453.4 ± 131.01	**Diet**	1.601	0.2141
						**Genotype**	2.852	0.1001
						**Diet X genotype**	7.034	**0.0119**
	Female	1830.4 ± 63.5	1764.6 ± 65.7	1909.2 ± 92.9	2069 ± 102.6	**Diet**	5.213	**0.0286**
						**Genotype**	0.313	0.5792
						**Diet X genotype**	1.807	0.1875
Total level of amino acids	Male	3175.7 ± 226.4	3166.7 ± 231.0	3366.01 ± 188.7	4337.2 ± 382.2	**Diet**	6.470	**0.0154**
						**Genotype**	3.235	0.0805
						**Diet X genotype**	3.357	0.0752
	Female	2846.02 ± 114.4	2844.3 ± 78.9	3023.8 ± 141.3	3366.3 ± 201.8	**Diet**	5.864	**0.0208**
						**Genotype**	1.386	0.2470
						**Diet X genotype**	1.431	0.2397
Ala/total amino acids	Male	0.1 ± 0.007	0.1 ± 0.005	0.1 ± 0.004	0.1 ± 0.004	**Diet**	0.918	0.3445
						**Genotype**	0.017	0.8984

						**Diet X genotype**	1.048	0.3129
	Female	0.09 ± 0.003	0.09 ± 0.002	0.1 ± 0.006	0.12 ± 0.004	**Diet**<br>**Genotype**<br>**Diet X genotype**	36.56<br>3.446<br>4.891	**<0.0001**<br>0.0718<br>**0.0336**
Gln/total amino acids	Male	0.17 ± 0.02	0.17 ± 0.01	0.15 ± 0.008	0.14 ± 0.01	**Diet**<br>**Genotype**<br>**Diet X genotype**	4.160<br>0.257<br>0.016	**0.0488**<br>0.6151<br>0.8993
	Female	0.2 ± 0.012	0.18 ± 0.009	0.19 ± 0.01	0.17 ± 0.01	**Diet**<br>**Genotype**<br>**Diet X genotype**	0.504<br>4.671<br>0.343	0.4827<br>**0.0376**<br>0.5622
Ala/essential amino acids	Male	0.3 ± 0.02	0.3 ± 0.02	0.3 ± 0.009	0.2 ± 0.02	**Diet**<br>**Genotype**<br>**Diet X genotype**	5.719<br>0.240<br>0.028	**0.0223**<br>0.6271<br>0.8676
	Female	0.3 ± 0.01	0.2 ± 0.01	0.3 ± 0.02	0.3 ± 0.01	**Diet**<br>**Genotype**<br>**Diet X genotype**	19.84<br>0.069<br>4.120	**<0.0001**<br>0.7938<br>**0.0500**
Gln/essential amino acids	Male	0.6 ± 0.07	0.5 ± 0.04	0.4 ± 0.03	0.4 ± 0.05	**Diet**<br>**Genotype**<br>**Diet X genotype**	14.98<br>1.278<br>0.574	**0.0005**<br>0.2660<br>0.4537
	Female	0.6 ± 0.05	0.5 ± 0.03	0.5 ± 0.05	0.4 ± 0.03	**Diet**<br>**Genotype**<br>**Diet X genotype**	0.450<br>5.573<br>0.062	0.5068<br>**0.0239**<br>0.8042
CPT 1	Male	0.01 ± 0.001	0.009 ± 0.001	0.017 ± 0.002	0.022 ± 0.002	**Diet**<br>**Genotype**<br>**Diet X genotype**	40.32<br>1.787<br>3.503	**<0.0001**<br>0.1899<br>0.0696
	Female	0.01 ± 0.002	0.011 ± 0.001	0.016 ± 0.001	0.020 ± 0.002	**Diet**<br>**Genotype**<br>**Diet X genotype**	29.36<br>0.0002<br>0.861	**<0.0001**<br>0.9876<br>0.3599
CPT 2	Male	0.02 ± 0.002	0.02 ± 0.001	0.04 ± 0.003	0.05 ± 0.004	**Diet**<br>**Genotype**<br>**Diet X genotype**	97.89<br>0.437<br>4.821	**<0.0001**<br>0.5129<br>**0.0346**
	Female	0.016 ± 0.0008	0.02 ± 0.0014	0.06 ± 0.005	0.06 ± 0.004	**Diet**<br>**Genotype**<br>**Diet X genotype**	145.3<br>0.631<br>0.401	**<0.0001**<br>0.4325<br>0.5306

SCAC/C0	Male	0.6 ± 0.04	0.7 ± 0.05	0.5 ± 0.02	0.6 ± 0.05	**Diet**	6.378	**0.0161**
						**Genotype**	2.765	0.1050
						**Diet X genotype**	0.161	0.6908
	Female	1.03 ± 0.1	0.7 ± 0.05	0.5 ± 0.04	0.5 ± 0.03	**Diet**	17.70	**0.0002**
						**Genotype**	3.664	0.0638
						**Diet X genotype**	4.348	**0.0444**
SCAC/BCAA	Male	0.03 ± 0.002	0.03 ± 0.003	0.01 ± 0.001	0.012 ± 0.002	**Diet**	55.62	**<0.0001**
						**Genotype**	0.444	0.5097
						**Diet X genotype**	0.475	0.4951
	Female	0.05 ± 0.007	0.03 ± 0.003	0.01 ± 0.001	0.011 ± 0.001	**Diet**	38.88	**<0.0001**
						**Genotype**	8.230	**0.0069**
						**Diet X genotype**	3.813	0.0589
