# Supplementary material for: High-Fat Diet Induces Pre-Diabetes and Distinct Sex-Specific Metabolic Alterations in Negr1-Deficient Mice
Source: Biomedicines. 2021 Sep 3;9(9):1148. doi: 10.3390/biomedicines9091148 (PMC8466019; doi:10.3390/biomedicines9091148)
Supplement: Supplementary file 1 [file biomedicines-09-01148-s001.zip › Supplementary table S2.pdf]

**Metabolites**	**Sex**	**Normal diet**		**HFD**		**Two-way ANOVA**		
		**Wild type** **Mean** ± SEM	**Negr1-/-** **Mean** ± SEM	**Wild type** **Mean** ± SEM	**Negr1-/-** **Mean** ± SEM		**F**	**p**
Malate	Male	9.9 ± 1.2	19.9 ± 3.7	7.1 ± 1.5	15.7 ± 2.8	**Diet**	2.1	0.1574
						**Genotype**	14.8	0.0007
						**Interaction**	0.08	0.7834
	Female	11.5 ± 2.8	17.1 ± 3.7	12.5 ± 2.3	9.7 ± 1.0	**Diet**	1.72	0.2023
						**Genotype**	0.34	0.5658
						**Interaction**	2.93	0.0998
Succinate	Male	9.6 ± 0.8	16.9 ± 2.9	8.5 ± 1.6	17.5 ± 2.7	**Diet**	0.01	0.9126
						**Genotype**	14.6	0.0007
						**Interaction**	0.15	0.7001
	Female	13.6 ± 2.6	16.1 ± 4.7	14.5 ± 2.4	10.6 ± 1.1	**Diet**	0.81	0.3781
						**Genotype**	0.07	0.7971
						**Interaction**	1.54	0.2272
Citrate	Male	168.5 ± 52.3	422.6 ± 168.1	112.3 ± 25.2	283.7 ± 83.5	**Diet**	1.16	0.2889
						**Genotype**	5.56	0.0256
						**Interaction**	0.21	0.6501
	Female	145.6 ± 42.2	377.6 ± 129.0	140.8 ± 65.1	206.5 ± 81.2	**Diet**	1.16	0.2918
						**Genotype**	3.32	0.0808
						**Interaction**	1.04	0.3183
Pyruvate	Male	47.0 ± 7.0	75.4 ± 7.0	41.0 ± 3.4	58.1 ± 8.1	**Diet**	2.97	0.0963
						**Genotype**	11.4	0.0022
						**Interaction**	0.70	0.4088
	Female	64.0 ± 11.6	55.0 ± 15.8	56.5 ± 11.5	48.7 ± 4.3	**Diet**	0.39	0.5407
						**Genotype**	0.58	0.4533
						**Interaction**	0.003	0.9562
AKG	Male	9.0 ± 1.5	17.0 ± 2.2	4.3 ± 0.6	10.5 ± 1.7	**Diet**	13.21	0.0012
						**Genotype**	21.59	0.0001
						**Interaction**	0.34	0.5639
	Female	9.7 ± 2.1	10.9 ± 2.3	9.1 ± 2.0	6.7 ± 0.7	**Diet**	1.58	0.2212
						**Genotype**	0.12	0.7283
						**Interaction**	0.91	0.3506
Lactate	Male	9.0 ± 1.5	14.6 ± 3.0	4.3 ± 0.6	10.5 ± 1.7	**Diet**	5.78	0.0233
						**Genotype**	10.61	0.0030
						**Interaction**	0.03	0.8709
	Female	9.7 ± 2.1	10.9 ± 2.3	9.1 ± 2.0	6.7 ± 0.7	**Diet**	1.58	0.2212
						**Genotype**	0.12	0.7283
						**Interaction**	0.91	0.3506
Oxaloacetate	Male	2.4 ± 0.3	4.3 ± 0.9	1.3 ± 0.4	4.1 ± 1.8	**Diet**	0.42	0.5224
						**Genotype**	4.95	0.0346
						**Interaction**	0.15	0.7003
	Female	6.0 ± 1.5	7.0 ± 2.6	4.6 ± 1.3	2.2 ± 0.3	**Diet**	5.02	0.0346
						**Genotype**	0.24	0.6258
						**Interaction**	1.45	0.2402

Beta-hydroxybutyrate	Male	38.4 ± 5.4	71.0 ± 13.9	26.0 ± 1.9	53.6 ± 8.0	**Diet**	3.50	0.0717
						**Genotype**	14.28	0.0007
						**Interaction**	0.10	0.7500
	Female	87.0 ± 11.0	72.4 ± 21.3	58.9 ± 12.6	59.5 ± 14.3	**Diet**	1.87	0.1843
						**Genotype**	0.22	0.6455
						**Interaction**	0.26	0.6159
